# Metabolomic profiling reveals key metabolites associated with hypertension progression

**DOI:** 10.3389/fcvm.2024.1284114

**Published:** 2024-02-08

**Authors:** Sarah Al Ashmar, Najeha Rizwana Anwardeen, Gulsen Guliz Anlar, Shona Pedersen, Mohamed A. Elrayess, Asad Zeidan

**Affiliations:** ^1^ Department of Basic Sciences, College of Medicine, QU Health, Qatar University, Doha, Qatar; ^2^ Biomedical Research Center, Qatar University, Doha, Qatar

**Keywords:** hypertension, pre-hypertension, metabolic profiling, biomarkers, disease progression

## Abstract

**Introduction:**

Pre-hypertension is a prevalent condition among the adult population worldwide. It is characterized by asymptomatic elevations in blood pressure beyond normal levels but not yet reaching the threshold for hypertension. If left uncontrolled, pre-hypertension can progress to hypertension, thereby increasing the risk of serious complications such as heart disease, stroke, kidney damage, and others.

**Objective:**

The precise mechanisms driving the progression of hypertension remain unknown. Thus, identifying the metabolic changes associated with this condition can provide valuable insights into potential markers or pathways implicated in the development of hypertension.

**Methods:**

In this study, we utilized untargeted metabolomics profiling, which examines over 1,000 metabolites to identify novel metabolites contributing to the progression from pre-hypertension to hypertension. Data were collected from 323 participants through Qatar Biobank.

**Results:**

By comparing metabolic profiles between pre-hypertensive, hypertensive and normotensive individuals, six metabolites including stearidonate, hexadecadienoate, N6-carbamoylthreonyladenosine, 9 and 13-S-hydroxyoctadecadienoic acid (HODE), 2,3-dihydroxy-5-methylthio- 4-pentenoate (DMTPA), and linolenate were found to be associated with increased risk of hypertension, in both discovery and validation cohorts. Moreover, these metabolites showed a significant diagnostic performance with area under curve >0.7.

**Conclusion:**

These findings suggest possible biomarkers that can predict the risk of progression from pre-hypertension to hypertension. This will aid in early detection, diagnosis, and management of this disease as well as its associated complications.

## 1 Introduction

Hypertension, a significant global health burden and a leading cause of premature death worldwide, affects approximately 1 billion people worldwide (1, 2). It is a critical risk factor for coronary heart disease and stroke, particularly in Gulf Cooperation Council (GCC) (3). In Qatar, hypertension prevalence is around 33% (4).

Pre-hypertension, an intermediate stage between normal and high blood pressure (5), affects 25 to 50% of adults globally (6). Recognized as a hypertension precursor (6), pre-hypertension can convert to hypertension at a rate of 30% over a four-year period (7) and contribute to cardiovascular disease progression (8). Cardiovascular risk factors, including genetics, smoking, obesity, and physical activity are linked to pre-hypertension (9, 10). Early detection of pre-hypertension can prevent hypertension and cardiovascular disease risk (11). Non-pharmacological interventions for the management of pre-hypertension include dietary, exercise, smoking cessation, reducing salt intake and alcohol consumption, weight, and stress reduction (6, 11, 12). If these measures fail, medication may be required (12). Hypertension management involves both non-pharmacological and pharmacological interventions including, antihypertensive drugs such as diuretics, angiotensin-converting enzyme inhibitors, angiotensin receptor blockers, beta-blockers, and calcium channel blockers (12, 13). However, despite the recent advances in the awareness, treatment, and management of hypertension, only 42% of hypertensive adults are diagnosed and treated, and only 21% have it under control (1).

Blood pressure changes are typically used to diagnose hypertension (14). However, many pathological changes precede blood pressure elevation (15). Therefore, identifying new biomarkers and understanding underlying mechanisms involved in hypertension progression is crucial. Metabolomics, a rapidly expanding field, involves the comprehensive analysis of all low-molecular-weight metabolites within biological samples at a given time (16). Metabolites play crucial roles in biological processes such as energy production, signaling, and cellular pathway regulation (16). Changes in these metabolites are being studied as potential biomarkers for cardiovascular disease and other pathological conditions (17). Metabolomics profiling can provide information about the metabolic changes in pathological conditions, potentially offering new insights into disease pathogenesis and therapeutic options (18). Clinically, metabolomics can aid in early disease detection and diagnosis (19). It also plays a significant role in precision medicine (20). By identifying unique metabolic signatures in individuals, it allows for personalized treatment strategies and guides for the selection of more effective treatments (20). Additionally, metabolomics helps in the discovery of biomarkers for various diseases, facilitating not only diagnosis but also monitoring disease progression and response to therapy (21). Recent advancements in metabolomics have significantly enhanced our understanding of hypertension (18). Metabolite profiling in hypertension has gained growing attention after the discovery of a correlation between serum metabolites and blood pressure in hypertensive individuals (17). Subsequently, numerous studies have focused on studying the metabolic signature of hypertension and identifying novel metabolites associated with this disease.

In this study, we aimed to characterize the metabolomic profile in pre-hypertensive and hypertensive patients and healthy individuals using untargeted metabolomics. We also examined the metabolites and the related metabolic pathways that can distinguish pre-hypertension from hypertension pathophysiology.

## 2 Materials and methods

### 2.1 Study participants

A total of 323 participants were enrolled in this study categorized based on their blood pressure. In the discovery cohort, 46 healthy controls, 98 patients with pre-hypertension, and 35 patients with hypertension were included, while the validation cohort included 72 healthy and 72 pre-hypertensive participants. Clinical and metabolomics data were provided by Qatar Biobank (QBB) which is a population-based cohort study launched in 2012 and aiming to recruit 60,000 local participants. 26,279 participants have been enrolled as of 2020. QBB collects personal data and biological samples for each participant every five years (22).

The inclusion criteria encompassed, both Qatari males and females, age between 46 and 68 years old for the discovery cohort, and between 20 and 34 for the validation cohort. The study groups comprised hypertensive participants (blood pressure ≥ 140 mmHg), pre-hypertensive (Blood pressure 120–139 mmHg), and a control group of normotensive participants (blood pressure < 120 mmHg). Patients with diabetes or obesity were excluded. An informed consent was obtained from each participant prior to the involvement in the study. The study received an approval from the QBB institutional review board (E -2021-QF-QBB-RES-ACC-00021-0160).

### 2.2 Sample collection

Approximately 60 ml of blood was collected from each participant following an overnight fasting. Biochemical and hematological assessment of the blood samples was conducted at Hamad Medical Center in Doha. Blood samples containing EDTA were centrifuged to separate various components, including plasma, buffy coat, and erythrocytes. Aliquots were prepared for each sample and stored either in liquid nitrogen for long-term storage or at −80°C for subsequent analysis (22).

### 2.3 Physical and clinical analysis

For each participant, anthropometric measurements such as the height, weight, waist, and hip were obtained using Seca stadiometer and Seca Bio Impedance Analysis (Seca GmbH & Co. KG, Hamburg, Germany). Body Mass Index was calculated as weight in kg divided by height in meters squared. Blood pressure was assessed utilizing the Omron 705 automated instrument. An average of two to three blood pressure measurements, taken when the initial two measurements differ by 5 mmHg, was considered. Clinical parameters, including complete blood count, lipid profile, HBA1C%, liver function tests and C-reactive protein were analyzed as previously described (22, 23).

### 2.4 Untargeted metabolomics analysis

Untargeted metabolomics was employed using Metabolon's platform at Anti-Doping Lab in Qatar to analyze the samples for each individual, following a previously described methodology (24). In brief, waters ACQUITY ultra-performance liquid chromatography (UPLC), a Thermo Scientific Q-Exactive high resolution/accurate mass spectrometer coupled with a heated electrospray ionization (HESI-II) source, and an Orbitrap mass analyzer with a 35,000-mass resolution were utilized for metabolite detection. Methanol was used for protein precipitation and serum sample extraction. The resulting extracts were partitioned into separate fractions: two for analysis by distinct reverse phases (RP)/UPLC-MS/MS methods using positive ion mode electrospray ionization (ESI), one for analysis by RP/UPLC-MS/MS with negative ion mode ESI, one for analysis by hydrophilic interaction chromatography (HILIC)/UPLC-MS/MS with negative ion mode ESI, and one aliquot reserved as a backup. Peaks identification was conducted using Metabolon's platform, matching them to existing library entries of over 3,300 pure standard chemicals to determine compound identities (25). Subsequently, compounds were classified based on their origins. A total of 1,159 metabolites were identified.

### 2.5 Statistical analysis

Clinical measurement data were classified into control, pre-hypertension, and hypertension groups according to their blood pressure. Data were presented as mean (SD), median (IQR) and number (percentage) for parametric, non-parametric and nominal variables respectively. Differences between the groups were tested by ANOVA/Kruskal–Wallis for parametric/non-parametric variables and Chi-square test for nominal variables. *Post-HOC* tests (Pairwise *T*-test/Dunnett's) were applied accordingly. A *p*-value significance level of 0.05 was used.

The metabolomics data were log-transformed. Multivariate analysis including unsupervised PCA (principal component analysis) and supervised OPLS-DA (orthogonal partial least square-discriminant analysis) were run using the software SIMCA® (version 16.0.1). Metabolites with >50% missingness and 222 unidentified metabolites were excluded from the models. R version 4.2.1 was used to perform linear models to identify significant metabolites differentiating the study group [control (0)—pre-hypertension (1)—hypertension (2), denoting disease progression]. The model also included the following confounders: age, gender, and BMI. Multiple testing correction method [False Discovery Rate (FDR)] was used to adjust the nominal *p*-values. FDR < 0.05 was considered statistically significant. ROC analysis for validation of the metabolites was performed using R package pROC. Spearman's correlation between clinical measurements and significant metabolites was carried out using R packages Hmisc and corrplot.

## 3 Results

### 3.1 General characteristics of the participants

In the discovery cohort, 178 participants were included: 46 control participants (blood pressure <120 mmHg), 98 participants with pre-hypertension (Blood pressure 120–139 mmHg) and 35 participants with hypertension (blood pressure ≥ 140 mmHg). As shown in Table 1, systolic and diastolic blood pressure measurements were significantly different between the three groups. The participants in control group were younger compared to pre-hypertensive and hypertensive groups and they were mostly females, however, no significant difference was observed between pre-hypertensive and hypertensive groups in terms of age and sex. Haemoglobin levels and red blood cells count were observed to be significantly lower in the control group compared to both the pre-hypertensive and hypertensive groups. This difference is mainly attributed to the predominance of females in the control group, as females typically exhibit lower haemoglobin and red blood cell levels. ALP, HDL cholesterol and triglyceride level also showed a significant difference between the control and disease groups; however, their values fell within normal levels. Chi square analysis further indicated no significant difference between pre-hypertensive and hypertensive participants taking blood pressuring lowering medications (Supplementary Table S1).

**Table 1:** Clinical measurements of the discovery cohort categorized by blood pressure.

Variable	Control (*N* = 46)	Pre-hypertension (*N* = 98)	Hypertension (*N* = 35)	*p*-value
Ethnicity	Qatari (100%)	Qatari (100%)	Qatari (100%)	
Sex				
Male	13 (28.26%)	53 (54.08%)	18 (51.43%)	**0**.**013**
Female	33 (71.74%)	45 (45.92%)	17 (48.57%)	
Age	48 (46.25–52)	50 (48–56)	55 (48.5–64)	**0**.**002**
BMI	26.28 (25.06–27.98)	27.46 (25.46–28.75)	27.11 (24.65–28.1)	0.060
Haemoglobin (g/dl)	12.95 (1.29)	13.83 (1.66)	14.03 (1.73)	**0**.**003**
Red blood cell ×10^6^/ul	4.6 (4.3–4.975)	5 (4.6–5.3)	4.9 (4.62–5.17)	**0**.**001**
White blood cell ×10^3^/ul	5.85 (4.72–6.9)	6.4 (5.4–7.125)	5.95 (4.9–6.7)	0.072
Glucose (mmol/l)	5.1 (4.6–5.6)	5.1 (4.8–5.4)	5.2 (4.8–5.79)	0.477
ALT (U/l)	20 (15–26.5)	21 (15–33)	19 (16–25.75)	0.494
AST (U/l)	19 (16.25–22)	19 (16–23)	18.5 (16–23.75)	0.899
ALP (U/l)	61 (53.25–69)	69 (59–80)	66.5 (58.25–78)	**0**.**030**
Cholesterol Total (mmol/l)	5.03 (0.67)	5.29 (1.08)	5.53 (1.13)	0.087
HDL Cholesterol (mmol/l)	1.665 (1.275–1.94)	1.31 (1.14–1.52)	1.45 (1.17–1.8)	**0**.**001**
LDL Cholesterol (mmol/l)	2.94 (0.69)	3.23 (0.97)	3.33 (1.00)	0.114
Triglyceride (mmol/l)	0.96 (0.71–1.11)	1.35 (1.1–1.8)	1.3 (1.015–1.71)	**4.55E-06**
HBA-1C%	5.5 (5.2–5.7)	5.5 (5.3–5.8)	5.7 (5.375–5.82)	0.269
C Reactive Protein (mg/l)	5 (5–5)	5 (5–5)	5 (5–5)	0.857
Average systolic BP	108.5 (104.25–115)	128 (123–133)	146 (143–154)	**4.03E-32**
Average diastolic BP	69 (64–75)	83 (77.25–86)	91 (83–99)	**9.67E-18**
History of other comorbidities				
Diabetes Mellitus	0	0	0	0.99
Obesity	0	0	0	0.99
Hypercholesterolemia	15 (32.6%)	52 (53%)	23 (65.7%)	**0**.**009**
Medications				
Blood pressure medications	0	13 (13.3%)	8 (22.8%)	**0**.**005**

Data are presented as mean (SD), median (IQR) and number (percentage) for parametric, non-parametric and nominal variables respectively. Differences between the groups were tested by ANOVA/Kruskal Wallis for parametric/non-parametric variables and Chi-square test for nominal variables. *Post-HOC* tests (Pairwise *T*-test/Dunnett's) were applied accordingly. A *p*-value significance level of 0.05 was used.

BMI, body mass index; ALT, alanine transaminase; AST, aspartate transaminase; ALP, alkaline phosphates; LDL, low density lipoprotein; HDL, high density lipoprotein.

The bold values are indicated *p*-value <0.05.

### 3.2 Metabolic profiling of healthy, pre-hypertensive and hypertensive participants (multivariant analysis)

**Figure 1:**
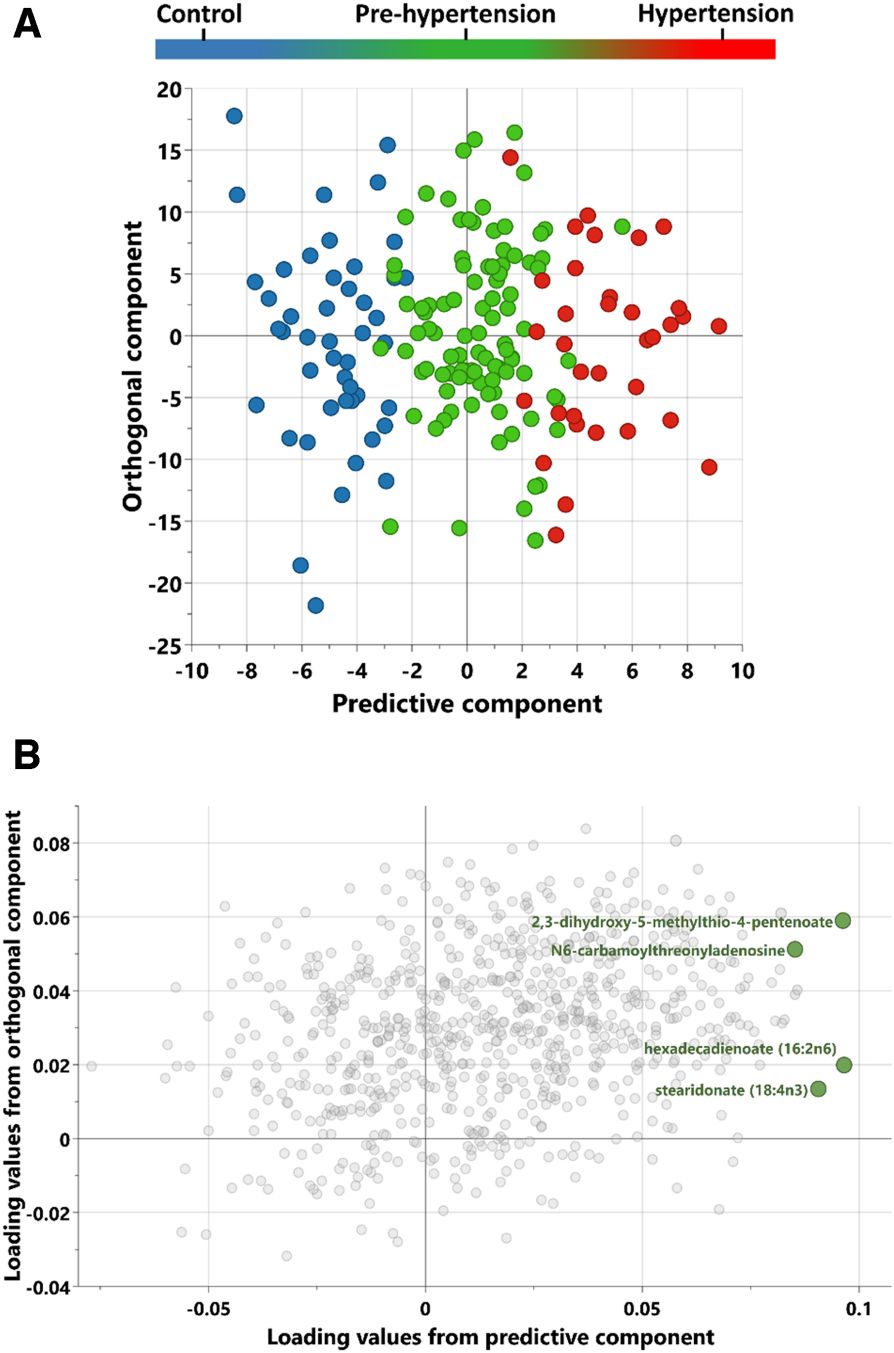
Scores and loadings plot from OPLS-DA (orthogonal projections to latent structures discriminant analysis) of control, pre-hypertension and hypertension discovery groups. (**A**) A score plot from OPLS-DA showing the predictive component (x-axis) vs. orthogonal component (y-axis). (**B**) Loadings plot revealing the discriminant metabolites associated with progression to hypertension.

The orthogonal partial least square discriminant analysis (OPLS-DA) was employed to evaluate the metabolites in our study. This analysis showed a clear separation between healthy, pre-hypertensive and hypertensive participants based on their metabolomic signature. The cumulative model predictability, R2Y (cum), was found to be 79.7%, while the cumulative model validation, Q2 (cum), was 22.3% indicating a strong ability to explain the observed patterns and an acceptable predictive accuracy. A scatter plot (Figure 1A) illustrates a two-dimensional space representation of the three study groups based on their metabolomic profiles. In addition to the scatter plot, we have provided supplementary data containing a list of the most influential metabolites for discriminating between the groups based on the variable influence on projection (VIP) scores (Supplementary Figure S1 and Table S2). Loading plot revealing the metabolites closely associated with the progression of hypertension is also presented in Figure 1B. Furthermore, to explore whether the metabolic profile vary between male and female participants, multivariate analysis was conducted for each gender separately. The OPLSDA for male participants demonstrated a distinct metabolic pattern for controls, prehypertensive, and hypertensive groups, achieving a high R2Y of 99% and a substantial predictive ability with Q2 of 48% (Supplementary Figure S2). Similarly, a clear group separation based on the metabolic profile was observed among female participants with 86% for R2Y and 22.6% for Q2 (Supplementary Figure S3). These results confirm that the observed group separation is primarily attributed to the disease condition rather than inherent sex differences.

### 3.3 Identification of the metabolites associated with hypertension progression (univariate analysis)

**Figure 2:**
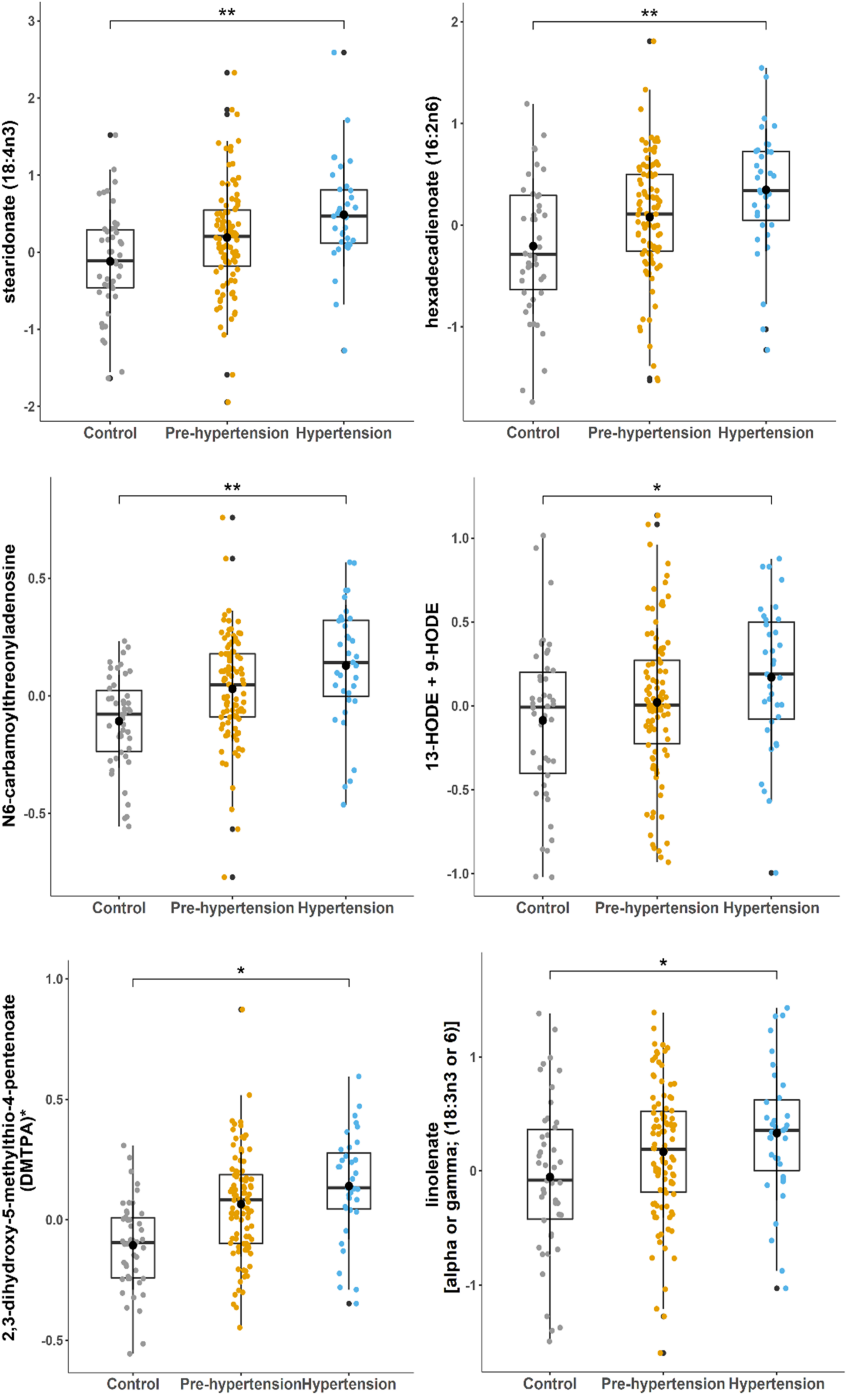
Boxplots of metabolites associated with hypertension progression in the discovery cohort. Linear regression performed using the R statistical package. Y-axis indicates levels of metabolites (ln). FDR significance level of 0.05 was used.

To identify the metabolites associated with increased risk of hypertension, a univariate analysis was conducted. Figure 2 displays the metabolites that were significantly correlated with an elevated risk of hypertension. These metabolites consist of four lipids {stearidonate [18:4n3], hexadecadienoate [16:2n6], 13-HODE + 9-HODE, and linolenate [alpha or gamma; (18:3n3 or 6)]}, one nucleotide (N6-carbamoylthreonyladenosine), and one amino acid metabolite (2,3-dihydroxy-5-methylthio-4-pentenoate (DMTPA) (Table 2). Moreover, these metabolites, associated with a higher risk of hypertension, exhibited nominal significance in the univariant analysis of males and females separately (Supplementary Tables S3, S4). These finding confirm the robustness and consistency of the association of the identified metabolites with hypertension risk across different sex groups.

**Table 2:** Metabolites significantly linked to a higher risk of hypertension in the discovery cohort.

Metabolites	Sub-pathway	Super-pathway	Estimate	Std Error	*p*-value	FDR
Stearidonate (18:4n3)	Lipid	Long chain polyunsaturated fatty acid (n3 and n6)	0.375	0.081	6.55E-06	0.003
Hexadecadienoate (16:2n6)	Lipid	Long chain polyunsaturated fatty acid (n3 and n6)	0.331	0.072	8.14E-06	0.003
N6-carbamoylthreonyladenosine	Nucleotide	Purine metabolism, adenine containing	0.092	0.025	3.04E-04	0.05
13-HODE + 9-HODE	Lipid	Fatty acid, monohydroxy	0.191	0.052	3.21E-04	0.05
2,3-dihydroxy-5-methylthio-4-pentenoate (DMTPA)	Amino Acid	Methionine, cysteine, SAM and taurine metabolism	0.080	0.022	3.66E-04	0.05
linolenate [alpha or gamma; (18:3n3 or 6)]	Lipid	Long chain polyunsaturated fatty acid (n3 and n6)	0.255	0.071	4.22E-04	0.05

### 3.4 Validation of the significant metabolites

**Figure 3:**
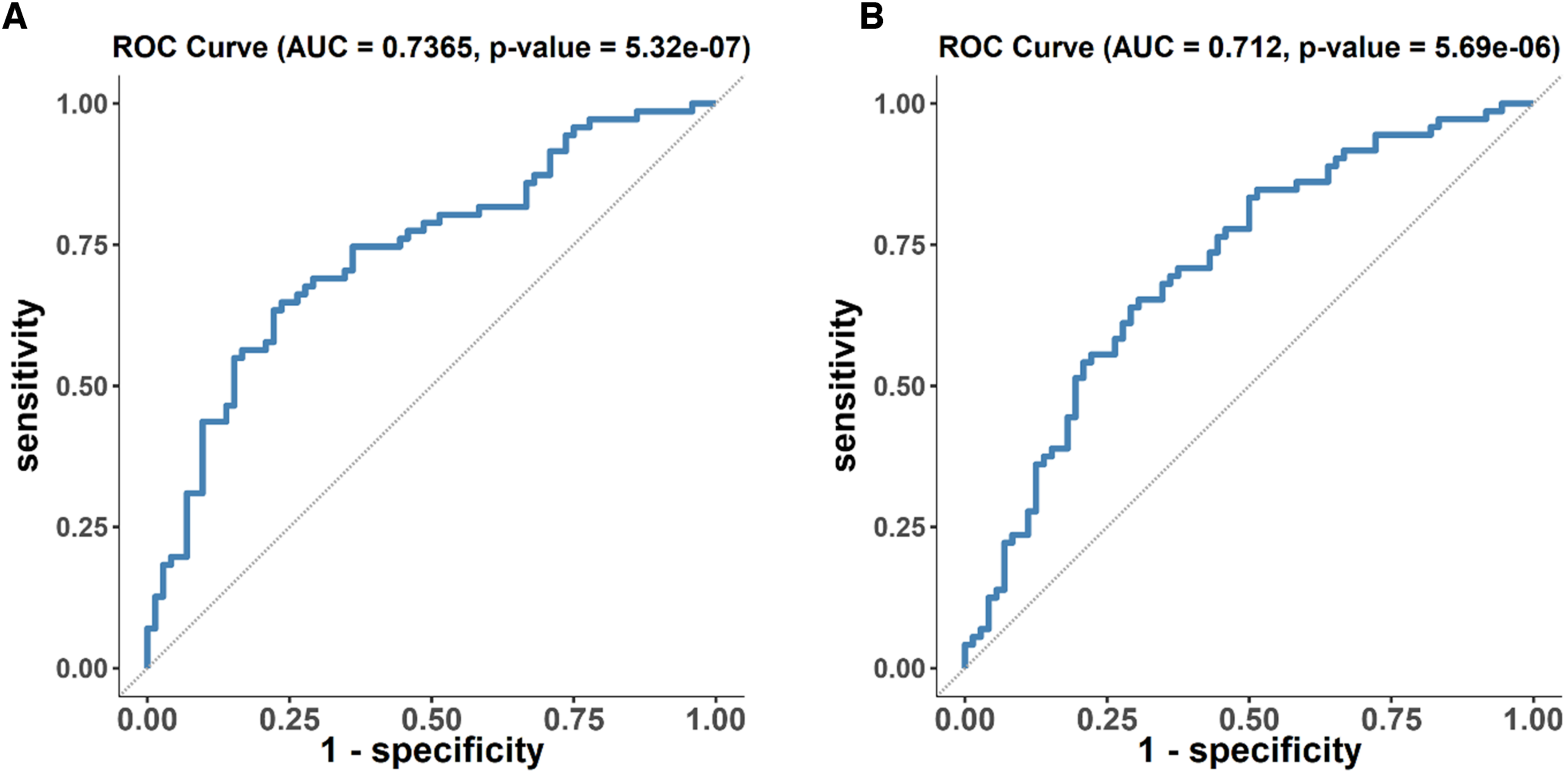
ROC analysis of metabolites associated with disease progression in the discovery cohort. (**A**) ROC analysis of six metabolites (stearidonate (18:4n3), hexadecadienoate (16:2n6), N6-carbamoylthreonyladenosine, 13-HODE + 9-HODE, 2,3-dihydroxy-5-methylthio-4-pentenoate (DMTPA), and linolenate [alpha or gamma; (18:3n3 or 6)]) (**B**) ROC analysis of 13-HODE + 9-HODE and 2,3-dihydroxy-5-methylthio-4-pentenoate (DMTPA) only.

In order to validate our findings, additional 72 healthy and 72 pre-hypertensive participants were used (table including their clinical profile is provided in Supplementary Table S5). By conducting a Receiver Operating Characteristic (ROC) analysis for the metabolites stearidonate (18:4n3), hexadecadienoate (16:2n6), N6-carbamoylthreonyladenosine, 13-HODE + 9-HODE, 2,3-dihydroxy-5-methylthio-4-pentenoate (DMTPA), and linolenate [alpha or gamma; (18:3n3 or 6)], we were able to determine their diagnostic performance. The cumulative ROC of all six metabolites provided AUC of 0.736 (Figure 3A) [95% CI: 0.654–0.818, *p*-value: 5.32 69 × 10-7]. However, the ROC of metabolites 13-HODE + 9-HODE and 2,3-dihydroxy-5-methylthio-4-pentenoate (DMTPA) provided AUC of 0.712 (Figure 3B) [95% CI: 0.627–0.796, *p*-value: 5.69 × 10-6], indicating a significant level of accuracy in predicting pre-hypertension risk, as evidenced by the significant *p*-value.

To strengthen our findings, further analysis was performed by combining the cohorts with diverse age ranges (below and above 40 years old). A random selection of 70% of the combined population was used as a discovery cohort, while the remaining 30% served as validation cohort. The obtained results confirmed our previously reported findings indicating a significant association between linolenate (18:3n3 or 6), stearidonate (18:4n3), hexadecadienoate (16:2n6), and 13-HODE + 9-HODE with an increased risk of hypertension progression in the new discovery cohort. Notably, stearidonate (18:4n3) and hexadecadienoate (16:2n6) also demonstrated significance in the validation cohort. The results of this analysis are included in Supplementary Table S6.

### 3.5 Correlation between the significant metabolites and clinical measurements in hypertensive patients

**Figure 4:**
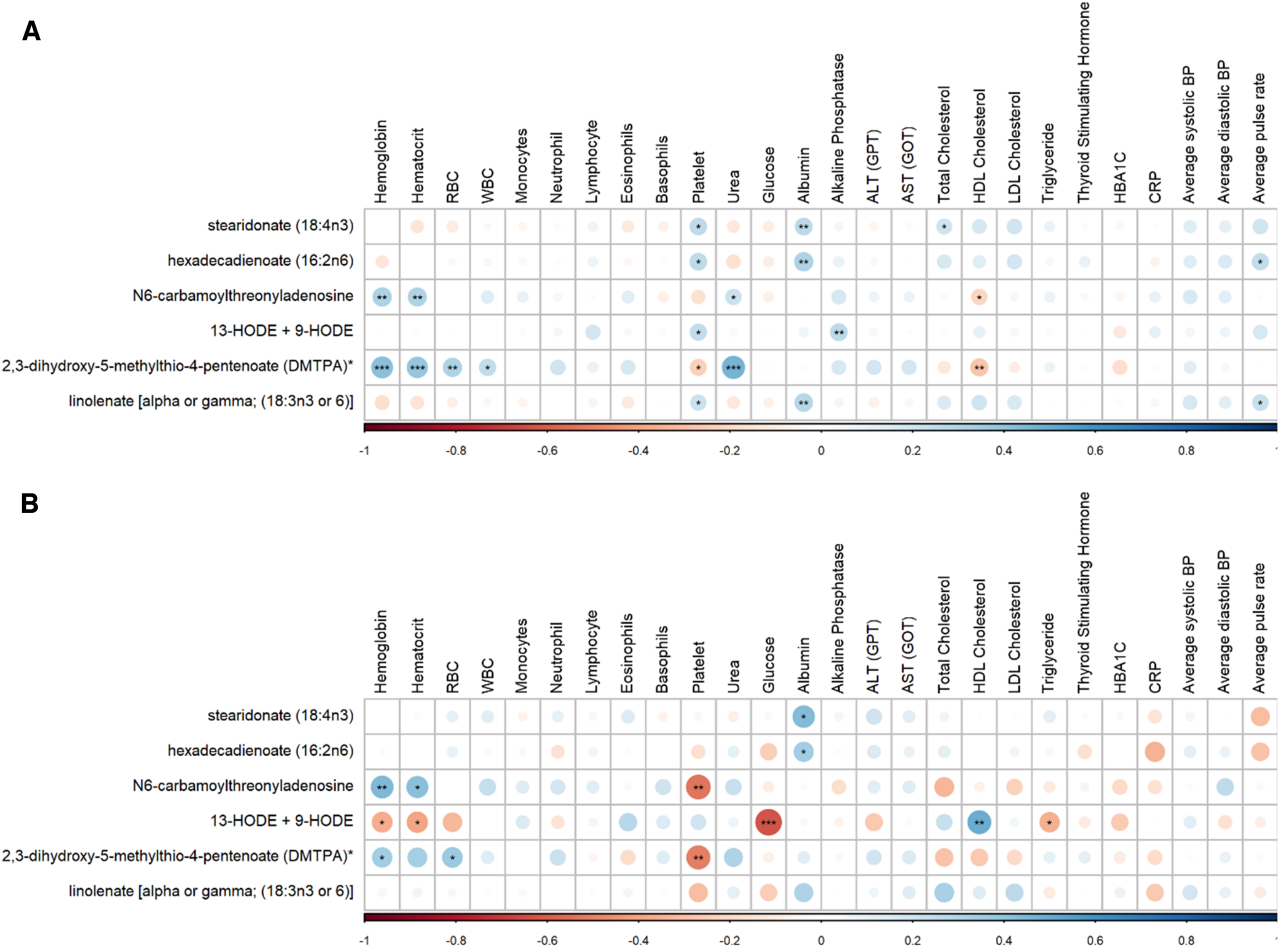
Correlation matrix between clinical characteristics and metabolites in (**A**) pre-hypertensive patients and (**B**) hypertensive discovery patients showing positive (blue) and negative (red) correlations. Significant correlations are displayed by ***/**/* denoting <0.001/<0.01/<0.05.

The results of the linear regression analysis' (Table 2) were used to undertake a correlation study between the clinical characteristics of pre-hypertensive and hypertensive patients and the significantly altered metabolites as shown in Figure 4.

## 4 Discussion

Epidemiological studies have reported that pre-hypertension is common worldwide (6), and it is likely to progress to hypertension and increase the risk of cardiovascular diseases (8). Metabolomic profiling has been widely used for the identification of novel pathways and biomarkers for hypertension (18), however, there is limited research on the metabolomic pathways underlying the progression of pre-hypertension to hypertension.

In this study, we have identified and validated numerous metabolites that exhibit significant association with an increased risk of hypertension in Qatari population. These metabolites play a crucial role in various metabolic pathways, providing insights into the underlying mechanisms contributing to the development and progression of this disease.

Our study revealed alterations in lipid metabolism, as evident from the changes in the levels of specific polyunsaturated fatty acids: stearidonate (18:4n-3), hexadecadienoate (16:2n6), and linolenate [alpha or gamma; (18:3n3 or 6)], between healthy, pre-hypertensive and hypertensive participants. Dysregulation of lipid metabolism has been previously linked to blood pressure elevation and cardiovascular risks, highlighting its importance in the context of hypertension (26–28). However, the direct relation between hypertension and stearidonate, hexadecadienoate, or linolenate, has not been established. First, in this study, we observed an elevation in stearidonate, which is an omega-3 fatty acid derived from *α*-linolenic acid (18:3 *n* -3) metabolic pathway (29, 30). This pathway has been shown to be implicated in many biological processes in the human body (30). Stearidonate has been found to be altered in many metabolomics studies. For example, metabolomics profiling of patients with excessive fluid retention, inflammation and acute graft vs. host rejection revealed an alteration in stearidonate (18:4n-3) (31). Exposure to dioxin, which is known to increase the risk of cardiovascular diseases and hypertension, also lead to increased stearidonate in mice liver (32). In addition, elevation in stearidonate (18:4n3) was noted in the metabolomic analysis of patients with argininosuccinate lyase deficiency (33). Plasma stearidonate levels were also elevated in untreated patients with primary dilated cardiomyopathy (34). Furthermore, stearidonate was significantly associated with increased urinary albumin excretion which indicates nephropathy and reflects endothelial dysfunction and kidney damage in the metabolomic analysis of patients with albuminuria (35). In contrast, several studies highlighted the potential benefits of stearidonate in relation to cardiovascular diseases (36, 37). It has been reported that stearidonate can exert an anti-thrombotic (30), anti-inflammatory (38, 39), as well as athero-protective effect (38). Second, our study revealed an alteration in hexadecadienoate (16:2n6) between healthy, pre-hypertensive and hypertensive participants. Hexadecadienoate is a long chain polyunsaturated fatty acid involved in lipid metabolism (40). Consistent with our results, a positive association between hexadecadienoate and systolic and diastolic blood pressure has reported in Chinese adults (41). Moreover, a study investigating the metabolomic profile in patients with chronic thromboembolic pulmonary hypertension showed higher hexadecadienoate levels in those patients compared to controls (31). Hexadecadienoate (16:2n6) was also listed among the metabolites differentiating insulin-sensitive from insulin-resistant lean/overweight subjects (42). In addition, analysis of the metabolic signature of leukocyte telomere length in elite soccer players, demonstrated that hexadecadienoate was associated with leukocyte telomere length in those individuals proposing a role of this metabolite in age-related diseases including cardiovascular diseases (43). Third, the polyunsaturated fatty acid linolenate (18:3n3 or 6) was shown to be associated with increased hypertension risk in the current study. Linolenate refers to either alpha-linolenic acid or gamma-linolenic acid (44). Alpha-linolenic acid is an essential omega-3 fatty acid highly present in edible and flaxseed oils (29), while gamma-linolenic acid is an omega-6 fatty acid, abundant in green leafy vegetables and nuts (45). Similar to our findings, a study comparing levels of metabolic predictors of coronary heart disease indicated that linolenate (18:3n3 or 6) was significantly different between lean and overweight females and was associated with elevated c-peptide and diastolic blood pressure (46). Zhe Wang et al. also reported that linoleante is associated with increased risk of coronary heart disease (47). Moreover, linolenate was found among the metabolites that differed significantly in dogs with dilated cardiomyopathy eating a non-traditional diet or a traditional diet (48). In addition, this metabolite was shown to be associated with urine albumin excretion rate (35). Together, these studies suggest a role for linoleante in hypertension and cardiovascular disease progression. On the other hand, clinical studies have also reported that alpha- linolenic acid can exert anti-obesity, anti-diabetic, anti-inflammatory, anti-cancer and anti-oxidize effects. It also has neuro and cardioprotective properties (49). Furthermore, food rich with alpha- linolenic acid was able to regulate blood pressure in hypertensive patients (49). Gamma- linolenic acid is also known for its potential benefits as an anti-inflammatory nutrient (45). Therefore, whether these polyunsaturated fatty acids are beneficial or deleterious to human health is still controversial and further research is needed to identify the role of these metabolite in the development of hypertension, as well as other pathological condition.

Moreover, our study uncovers the importance of purine metabolism disturbances, with N6-carbamoylthreonyladenosine, a nucleotide metabolite, significantly associated with increased risk of hypertension. Consistent with our finding, it has been reported that N6-carbamoylthreonyladenosine was significantly associated with systolic blood pressure (41). It has been identified as a biomarker for chronic kidney disease progression in children (50, 51) and highly correlated with glomerular filtration rate (52). Hypertension can often lead to end organ damage including renal dysfunction and chronic kidney disease (53). In addition, chronic kidney disease patients often face a higher risk of cardiovascular complications, including hypertension (54, 55). N6-carbamoylthreonyladenosine was shown to be associated with inflammatory marker IL-6 in older adults (56). Hypertension has been linked to inflammation and hypertensive patients have higher levels of IL-6 (57, 58). Thus, investigating the effect of N6-carbamoylthreonyladenosine on blood pressure regulation is important for early detection and prevention of hypertension, particularly in patients with chronic kidney disease.

Additionally, this study identified 13-HODE and 9-HODE as biomarkers for the progression of hypertension. 13-HODE and 9-HODE, known as oxylipins, are oxidized metabolites of linoleic acid which is the most common polyunsaturated fatty acid found in human diets (59). Several studies have demonstrated the contribution of oxylipins, in general, to cardiovascular diseases including hypertension (60), however, limited studies are available on the influence of 13-HODE and 9-HODE, specifically, on hypertension. A study have reported a significant increase in serum 13-HODE in patients with essential hypertension and it was positively correlated with mean blood pressure (61). Another study suggested that 13-HODE and 9-HODE are associated with pulmonary arterial hypertension (32). 9 and 13-HODE were considered among the most important derivatives after an early incident of ischemic stroke (62). Furthermore, 13-HODE and 9-HODE can affect vascular cells including smooth muscle cells (62). Alteration in vascular smooth muscle cell signaling and function can affect vascular reactivity and tone, which are critical determinants of vascular resistance and blood pressure (63). Several studies also show that HODE can induce pro-inflammatory effect including the production of inflammatory cytokines IL-1β and IL-8 (64, 65),and the activation of NF-*κ*B (66). In addition, 13-HODE and 9-HODE have been considered as biomarkers for oxidative stress and are linked to various pathological conditions such as atherosclerosis, diabetes, chronic inflammation, obesity, and cancer (59, 67). As oxidative stress plays an important role in the progression of hypertension, the strong relationship between 13-HODE and 9-HODE and oxidative stress (68) further emphasizes the potential role of those metabolites in the progression of this disease.

Finally, our results showed alteration in 2,3-dihydroxy-5-methylthio-4-pentenoate (DMTPA) between the study groups. DMTPA is an amino acid metabolite derived from S-adenosyl methionine (69). This metabolite is considered as uremic toxin and increased in patients with acute kidney injury (69). A study have revealed an association between DMTPA and systolic blood pressure (41). It has also been shown to be significantly correlated with BMI (70). High BMI can increase the risk of hypertension (71). Although the direct role of DMTPA in hypertension remains unclear, its connection to renal function suggests a possible link. Further research is needed to better understand the role of DMTPA in the context of hypertension.

To our knowledge, this is the first study identifying the metabolites associated with hypertension progression in Qatar. Despite the fact that our study provides significant insights, we acknowledge that certain limitations may have affected the outcomes. First, the data provided by Qatar Biobank did not specify whether the patients included in the study had primary or secondary hypertension. These two types might have distinct metabolic signatures in hypertensive patients. Second, the absence of hypertensive participants for validation due to limited data availability. However, validation in pre-hypertensive participants can still indicate that these metabolites might increase the risk of hypertension progression in these individuals. Thirdly, the study lacks information on the menopausal stage of female participants, especially given the age range of the discovery cohort (40–65 years), indicative of pre-menopausal or menopausal stages. Menopause, being associated with hypertension, might pose a potential confounding factor in this study.

In conclusion, in this study we have identified a set of metabolites associated with the progression of hypertension. Our study is exploratory in nature, aiming to shed light on the metabolic changes associated with hypertension. It serves as a foundation for further research necessary for the potential integration of metabolomics into clinical practice. Furthermore, our findings contribute to the development of novel diagnostic and predictive tools that could transform the way we approach and manage hypertension in the future.

## Data Availability

The datasets presented in this study can be found in online in an online repository: https://doi.org/10.6084/m9.figshare.25093178.v1.
