# Supplementary material for: Metabolomic profiling reveals key metabolites associated with hypertension progression
Source: Front Cardiovasc Med. 2024 Feb 8;11:1284114. doi: 10.3389/fcvm.2024.1284114 (PMC10881871; doi:10.3389/fcvm.2024.1284114)
Supplement: Supplementary file 1 [file Table1.docx]

Supplementary Material

Metabolomic Profiling Reveals Key Metabolites Associated with Hypertension Progression in Qatar

**Sarah Al Ashmar^1^, Najeha Anwardeen^2^, Gulsen Guliz Anlar^1^, Shona Pedersen^1^, Mohamed A. Elrayess^1,2^ and Asad Zeidan^1*^**

^1^ Department of Basic Sciences, College of Medicine, QU Health, Qatar University, Doha, Qatar.

^2^ Biomedical Research Center, Qatar University, Doha, Qatar.

*** Correspondence:**

Dr. Asad Zeidan, Ph.D.

Department of Basic Sciences, College of Medicine,

QU Health, P.O. Box 2713

Doha, Qatar,

[a.zeidan@qu.edu.qa](mailto:a.zeidan@qu.edu.qa)

**Supplementary Table S1:** Clinical characteristics of the discovery cohort categorized by blood pressure. Differences between the groups were tested by ANOVA/Kruskal Wallis for parametric/non-parametric variables and Chi-square test for nominal variables. Post-HOC tests (Pairwise T-test/Dunnett’s) were applied accordingly.

| **Variable** | **Control (N=46)** | **Pre-hypertension (N=98)** | | **Hypertension (N=35)** | **p-value** | **Control vs Pre-hypertension** | **Control vs Hypertension** | **Pre-hypertension vs Hypertension** |
| --- | --- | --- | --- | --- | --- | --- | --- | --- |
| **Ethnicity** | Qatari | Qatari | Qatari | |  |  |  |  |
| **Sex** |  |  |  | |  |  |  |  |
| Male | 13 | 53 | 18 | | **0.013** | **0.006** | 0.058 | 0.942 |
| Female | 33 | 45 | 17 | |  |  |  |  |
| **Age** | 48 | 50 | 55 | | **0.002** | **0.037** | **0.001** | **0.037** |
| **BMI** | 26.28 | 27.46 | 27.11 | | 0.06 | 0.087 | 0.681 | 0.195 |
| **Hemoglobin (g/dl)** | 12.95 | 13.83 | 14.03 | | **0.003** | **0.007** | **0.008** | 0.791 |
| **RBC x10^6^/ul** | 4.6 | 5 | 4.9 | | **0.001** | **0.001** | **0.011** | 0.907 |
| **WBC x10^3^/ul** | 5.85 | 6.4 | 5.95 | | 0.072 | 0.119 | 0.809 | 0.879 |
| **Glucose (mmol/l)** | 5.1 | 5.1 | 5.2 | | 0.477 | 0.688 | 0.488 | 0.488 |
| **ALT (U/L)** | 20 | 21 | 19 | | 0.494 | 0.684 | 0.684 | 0.684 |
| **AST (U/L)** | 19 | 19 | 18.5 | | 0.899 | 0.895 | 0.895 | 0.895 |
| **ALP (U/L)** | 61 | 69 | 66.5 | | **0.03** | **0.024** | 0.213 | 0.474 |
| **Total Cholesterol (mmol/l)** | 5.03 | 5.29 | 5.53 | | 0.087 | 0.325 | 0.073 | 0.448 |
| **HDL (mmol/l)** | 1.665 | 1.31 | 1.45 | | **0.001** | **0.001** | 0.148 | 0.148 |
| **LDL (mmol/l)** | 2.94 | 3.23 | 3.33 | | 0.114 | 0.176 | 0.148 | 0.859 |
| **Triglyceride (mmol/l)** | 0.96 | 1.35 | 1.3 | | **4.55E-06** | **3.99E-06** | **0.001** | 0.735 |
| **HBA-1C%** | 5.5 | 5.5 | 5.7 | | 0.269 | 0.691 | 0.25 | 0.25 |
| **C Reactive Protein (mg/l)** | 5 | 5 | 5 | | 0.857 | 0.867 | 0.867 | 0.867 |
| **Average systolic BP** | 108.5 | 128 | 146 | | **4.03E-32** | **1.10E-14** | **2.72E-32** | **7.01E-11** |
| **Average diastolic BP** | 69 | 83 | 91 | | **9.67E-18** | **5.78E-11** | **4.34E-17** | **2.00E-04** |
| **History of other comorbidities** |  |  |  | |  |  |  |  |
| Diabetes Mellitus | 0 | 0 | 0 | | 0.99 | 0.999 | 0.999 | 0.999 |
| Obesity | 0 | 0 | 0 | | 0.99 | 0.999 | 0.999 | 0.999 |
| Hypercholesterolemia | 15 | 52 | 23 | | **0.009** | **0.03** | **0.004** | 0.236 |
| **Medications** |  |  |  | |  |  |  |  |
| Blood pressure medications | 0 | 13 | 8 | | **0.005** | **0.01** | **0.001** | 0.187 |
|  |  |  |  | |  |  |  |  |

#
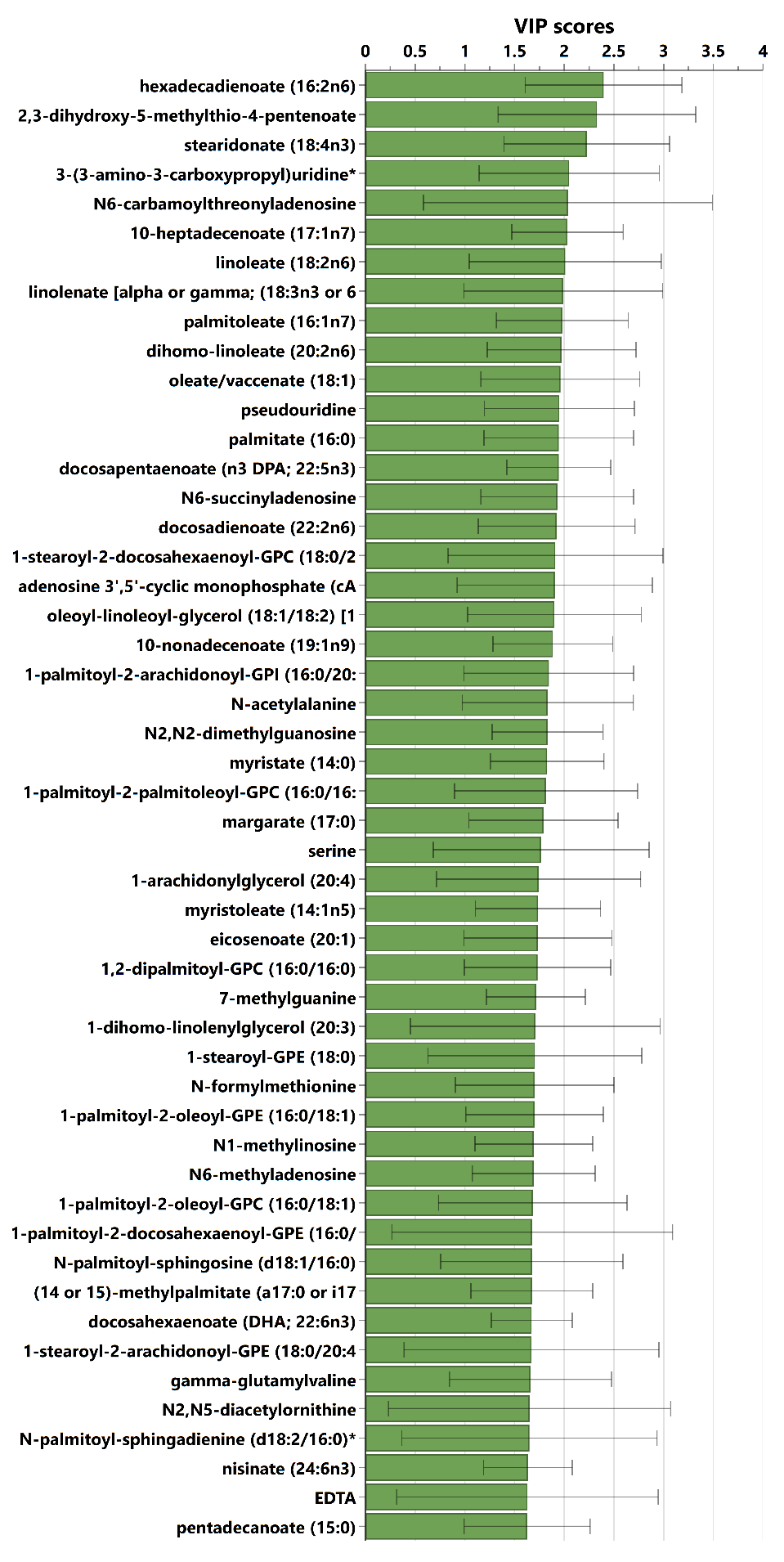


# Supplementary Figure S1: VIP list of top 50 metabolites from OPLS-DA model for the discovery cohort.

# Supplementary Table S2: VIP scores of the top 50 metabolites from OPLS-DA analysis for the discovery cohort.

| BIOCHEMICAL | SUB PATHWAY | SUPER PATHWAY | VIP |
| --- | --- | --- | --- |
| hexadecadienoate (16:2n6) | Long Chain Polyunsaturated Fatty Acid (n3 and n6) | Lipid | 2.39657 |
| 2,3-dihydroxy-5-methylthio-4-pentenoate (DMTPA) | Methionine, Cysteine, SAM and Taurine Metabolism | Amino Acid | 2.32896 |
| stearidonate (18:4n3) | Long Chain Polyunsaturated Fatty Acid (n3 and n6) | Lipid | 2.22683 |
| 3-(3-amino-3-carboxypropyl)uridine | Pyrimidine Metabolism, Uracil containing | Nucleotide | 2.05074 |
| N6-carbamoylthreonyladenosine | Purine Metabolism, Adenine containing | Nucleotide | 2.03718 |
| 10-heptadecenoate (17:1n7) | Long Chain Monounsaturated Fatty Acid | Lipid | 2.03256 |
| linoleate (18:2n6) | Long Chain Polyunsaturated Fatty Acid (n3 and n6) | Lipid | 2.00945 |
| linolenate [alpha or gamma; (18:3n3 or 6)] | Long Chain Polyunsaturated Fatty Acid (n3 and n6) | Lipid | 1.99005 |
| palmitoleate (16:1n7) | Long Chain Monounsaturated Fatty Acid | Lipid | 1.98098 |
| dihomo-linoleate (20:2n6) | Long Chain Polyunsaturated Fatty Acid (n3 and n6) | Lipid | 1.9735 |
| oleate/vaccenate (18:1) | Long Chain Monounsaturated Fatty Acid | Lipid | 1.96149 |
| pseudouridine | Pyrimidine Metabolism, Uracil containing | Nucleotide | 1.95109 |
| palmitate (16:0) | Long Chain Saturated Fatty Acid | Lipid | 1.94563 |
| docosapentaenoate (n3 DPA; 22:5n3) | Long Chain Polyunsaturated Fatty Acid (n3 and n6) | Lipid | 1.94559 |
| N6-succinyladenosine | Purine Metabolism, Adenine containing | Nucleotide | 1.92951 |
| docosadienoate (22:2n6) | Long Chain Polyunsaturated Fatty Acid (n3 and n6) | Lipid | 1.92368 |
| 1-stearoyl-2-docosahexaenoyl-GPC (18:0/22:6) | Phosphatidylcholine (PC) | Lipid | 1.91209 |
| adenosine 3',5'-cyclic monophosphate (cAMP) | Purine Metabolism, Adenine containing | Nucleotide | 1.90589 |
| oleoyl-linoleoyl-glycerol (18:1/18:2) [1] | Diacylglycerol | Lipid | 1.90177 |
| 10-nonadecenoate (19:1n9) | Long Chain Monounsaturated Fatty Acid | Lipid | 1.88567 |
| 1-palmitoyl-2-arachidonoyl-GPI (16:0/20:4)* | Phosphatidylinositol (PI) | Lipid | 1.84527 |
| N-acetylalanine | Alanine and Aspartate Metabolism | Amino Acid | 1.83446 |
| N2,N2-dimethylguanosine | Purine Metabolism, Guanine containing | Nucleotide | 1.83129 |
| myristate (14:0) | Long Chain Saturated Fatty Acid | Lipid | 1.82718 |
| 1-palmitoyl-2-palmitoleoyl-GPC (16:0/16:1)* | Phosphatidylcholine (PC) | Lipid | 1.81639 |
| margarate (17:0) | Long Chain Saturated Fatty Acid | Lipid | 1.79128 |
| serine | Glycine, Serine and Threonine Metabolism | Amino Acid | 1.76768 |
| 1-arachidonylglycerol (20:4) | Monoacylglycerol | Lipid | 1.74187 |
| myristoleate (14:1n5) | Long Chain Monounsaturated Fatty Acid | Lipid | 1.7358 |
| eicosenoate (20:1) | Long Chain Monounsaturated Fatty Acid | Lipid | 1.73457 |
| 1,2-dipalmitoyl-GPC (16:0/16:0) | Phosphatidylcholine (PC) | Lipid | 1.73104 |
| 7-methylguanine | Purine Metabolism, Guanine containing | Nucleotide | 1.71612 |
| 1-dihomo-linolenylglycerol (20:3) | Monoacylglycerol | Lipid | 1.70806 |
| 1-stearoyl-GPE (18:0) | Lysophospholipid | Lipid | 1.70399 |
| N-formylmethionine | Methionine, Cysteine, SAM and Taurine Metabolism | Amino Acid | 1.70337 |
| 1-palmitoyl-2-oleoyl-GPE (16:0/18:1) | Phosphatidylethanolamine (PE) | Lipid | 1.70133 |
| N1-methylinosine | Purine Metabolism, (Hypo)Xanthine/Inosine containing | Nucleotide | 1.69308 |
| N6-methyladenosine | Purine Metabolism, Adenine containing | Nucleotide | 1.69244 |
| 1-palmitoyl-2-oleoyl-GPC (16:0/18:1) | Phosphatidylcholine (PC) | Lipid | 1.68278 |
| 1-palmitoyl-2-docosahexaenoyl-GPE (16:0/22:6) | Phosphatidylethanolamine (PE) | Lipid | 1.67795 |
| N-palmitoyl-sphingosine (d18:1/16:0) | Ceramides | Lipid | 1.67471 |
| (14 or 15)-methylpalmitate (a17:0 or i17:0) | Fatty Acid, Branched | Lipid | 1.67381 |
| docosahexaenoate (DHA; 22:6n3) | Long Chain Polyunsaturated Fatty Acid (n3 and n6) | Lipid | 1.67355 |
| 1-stearoyl-2-arachidonoyl-GPE (18:0/20:4) | Phosphatidylethanolamine (PE) | Lipid | 1.67031 |
| gamma-glutamylvaline | Gamma-glutamyl Amino Acid | Peptide | 1.66097 |
| N2,N5-diacetylornithine | Urea cycle; Arginine and Proline Metabolism | Amino Acid | 1.6521 |
| N-palmitoyl-sphingadienine (d18:2/16:0) | Ceramides | Lipid | 1.64928 |
| nisinate (24:6n3) | Long Chain Polyunsaturated Fatty Acid (n3 and n6) | Lipid | 1.63434 |
| EDTA | Chemical | Xenobiotics | 1.63065 |
| pentadecanoate (15:0) | Long Chain Saturated Fatty Acid | Lipid | 1.62578 |
| 3-hydroxybutyroylglycine | Fatty Acid Metabolism (Acyl Glycine) | Lipid | 1.62397 |
| palmitoylcarnitine (C16) | Fatty Acid Metabolism (Acyl Carnitine, Long Chain Saturated) | Lipid | 1.62111 |
| N-stearoyl-sphingosine (d18:1/18:0) | Ceramides | Lipid | 1.61648 |
| 1-linoleoylglycerol (18:2) | Monoacylglycerol | Lipid | 1.60048 |
| erythritol | Food Component/Plant | Xenobiotics | 1.59926 |
| 1-palmitoyl-GPE (16:0) | Lysophospholipid | Lipid | 1.59637 |
| hydroxyasparagine | Alanine and Aspartate Metabolism | Amino Acid | 1.58821 |
| 5-dodecenoate (12:1n7) | Medium Chain Fatty Acid | Lipid | 1.58317 |
| thioproline | Chemical | Xenobiotics | 1.57856 |
| tetradecadienoate (14:2) | Long Chain Polyunsaturated Fatty Acid (n3 and n6) | Lipid | 1.57388 |
| stearate (18:0) | Long Chain Saturated Fatty Acid | Lipid | 1.56714 |
| (16 or 17)-methylstearate (a19:0 or i19:0) | Fatty Acid, Branched | Lipid | 1.56554 |
| 1-palmitoyl-2-arachidonoyl-GPE (16:0/20:4) | Phosphatidylethanolamine (PE) | Lipid | 1.55814 |
| indolelactate | Tryptophan Metabolism | Amino Acid | 1.55153 |
| oleoyl ethanolamide | Endocannabinoid | Lipid | 1.54961 |
| alpha-CMBHC glucuronide | Tocopherol Metabolism | Cofactors and Vitamins | 1.54681 |
| 16-hydroxypalmitate | Fatty Acid, Monohydroxy | Lipid | 1.54324 |
| 2-O-methylascorbic acid | Ascorbate and Aldarate Metabolism | Cofactors and Vitamins | 1.53236 |
| N-acetylcarnosine | Histidine Metabolism | Amino Acid | 1.5313 |
| glucuronate | Aminosugar Metabolism | Carbohydrate | 1.52934 |
| dodecadienoate (12:2) | Fatty Acid, Dicarboxylate | Lipid | 1.52462 |
| branched-chain, straight-chain, or cyclopropyl 12:1 fatty acid | Partially Characterized Molecules | Partially Characterized Molecules | 1.5221 |
| N,N,N-trimethyl-alanylproline betaine (TMAP) | Urea cycle; Arginine and Proline Metabolism | Amino Acid | 1.522 |
| 1-palmitoyl-2-docosahexaenoyl-GPC (16:0/22:6) | Phosphatidylcholine (PC) | Lipid | 1.52131 |
| glycerol | Glycerolipid Metabolism | Lipid | 1.51263 |
| 7-alpha-hydroxy-3-oxo-4-cholestenoate (7-Hoca) | Sterol | Lipid | 1.50954 |
| gamma-glutamylphenylalanine | Gamma-glutamyl Amino Acid | Peptide | 1.5042 |
| 13-HODE + 9-HODE | Fatty Acid, Monohydroxy | Lipid | 1.50068 |
| HWESASXX | Polypeptide | Peptide | 1.50036 |

**
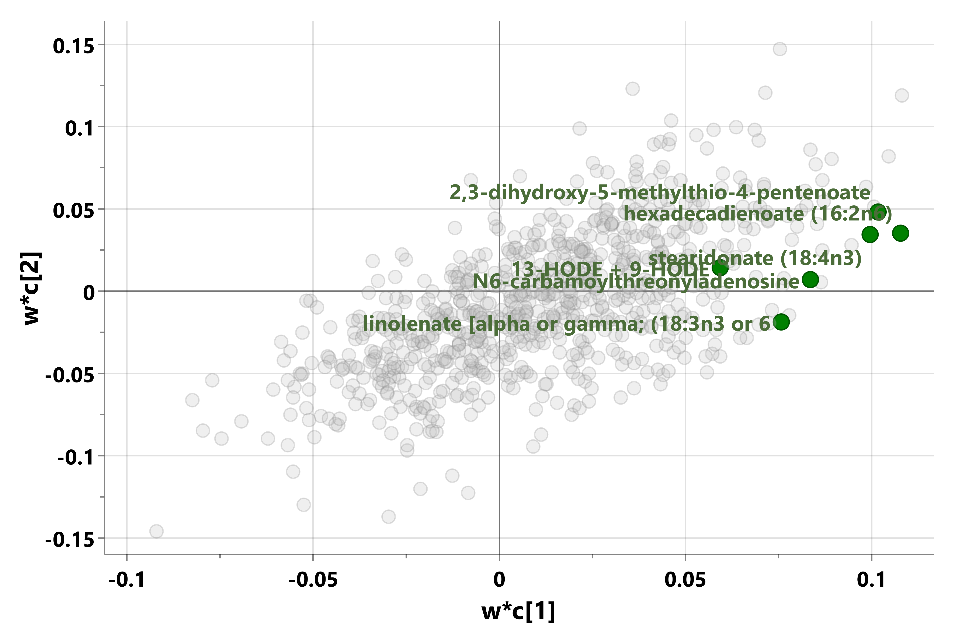

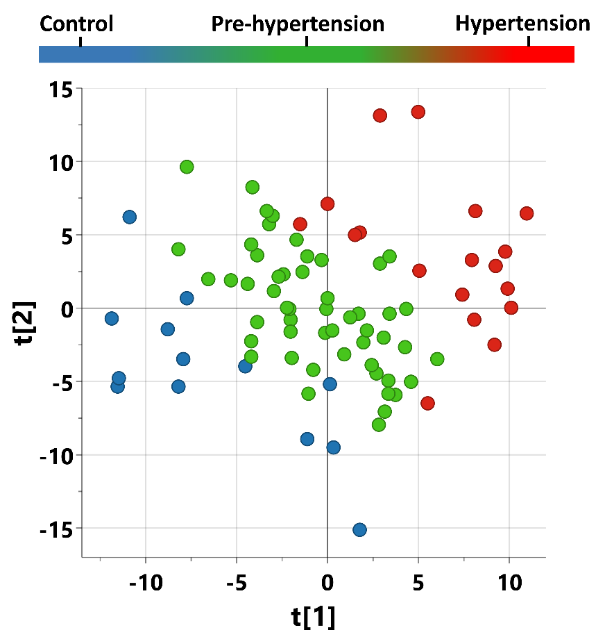
**

(a)

(b)

**Supplementary Figure S2:** Scores plot (a) and correspond plot (b) from the OPLS-DA performed on male participants from the discovery cohort (R2X = 31.5%, R2Y = 99%, Q2 = 48%). The significant biomarkers of interest are highlighted in the loadings plot.


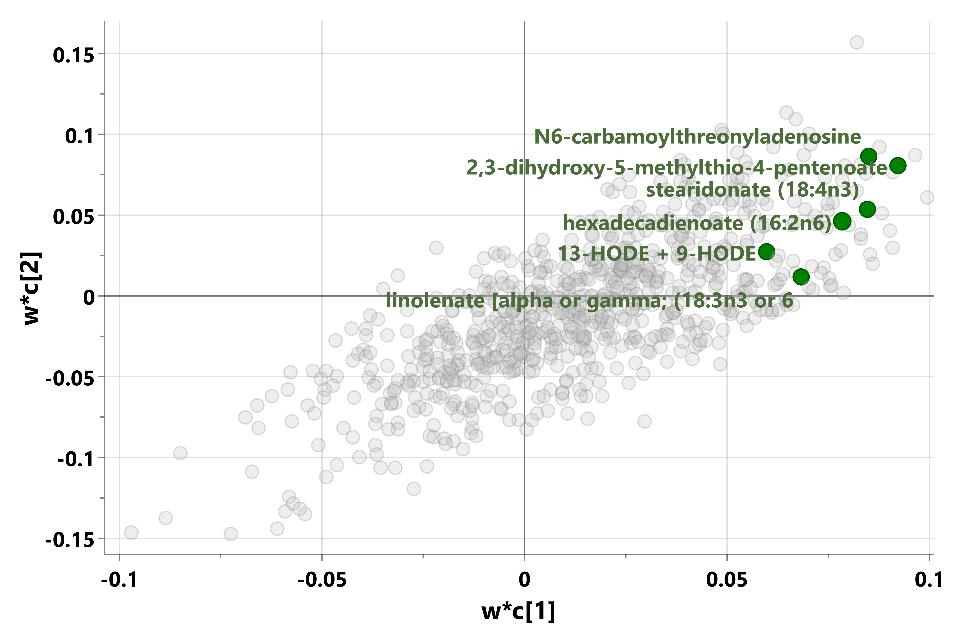

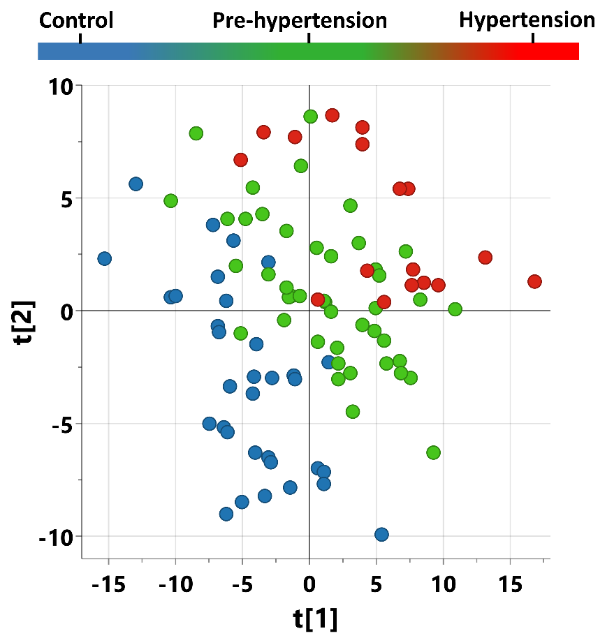


(b)

(a)

**Supplementary Figure S3:** Scores plot (a) and correspond plot (b) from the OPLS-DA performed on female participants from the discovery cohort (R2X = 16%, R2Y = 86%, Q2 = 22.6%). The significant biomarkers of interest are highlighted in the loadings plot.

**Supplementary Table S3:** Metabolites linked to higher risk of hypertension in males.

| **Metabolites** | **Super-pathway** | | **Sub-pathway** | **Estimate** | **SE** | **p-value** | **FDR** |
| --- | --- | --- | --- | --- | --- | --- | --- |
| **hexadecadienoate (16:2n6)** | | **Lipid** | **Long Chain Polyunsaturated Fatty Acid (n3 and n6)** | **0.430** | **0.098** | **0.000** | **0.031** |
| **stearidonate (18:4n3)** | | **Lipid** | **Long Chain Polyunsaturated Fatty Acid (n3 and n6)** | **0.429** | **0.112** | **0.000** | **0.103** |
| N-acetylcarnosine | | Amino Acid | Histidine Metabolism | 0.201 | 0.062 | 0.002 | 0.303 |
| erythritol | | Xenobiotics | Food Component/Plant | 0.155 | 0.049 | 0.002 | 0.303 |
| serine | | Amino Acid | Glycine, Serine and Threonine Metabolism | -0.092 | 0.029 | 0.002 | 0.303 |
| indolelactate | | Amino Acid | Tryptophan Metabolism | 0.106 | 0.034 | 0.002 | 0.303 |
| betonicine | | Xenobiotics | Food Component/Plant | -0.971 | 0.318 | 0.004 | 0.303 |
| 2-hydroxyhippurate (salicylurate) | | Xenobiotics | Benzoate Metabolism | 0.624 | 0.209 | 0.004 | 0.303 |
| gamma-glutamylmethionine | | Peptide | Gamma-glutamyl Amino Acid | -0.128 | 0.043 | 0.004 | 0.303 |
| N6-methyladenosine | | Nucleotide | Purine Metabolism, Adenine containing | 0.095 | 0.032 | 0.004 | 0.303 |
| 1-(1-enyl-stearoyl)-2-linoleoyl-GPE (P-18:0/18:2)* | | Lipid | Plasmalogen | -0.202 | 0.069 | 0.005 | 0.303 |
| N1-methylinosine | | Nucleotide | Purine Metabolism, (Hypo)Xanthine/Inosine containing | 0.146 | 0.050 | 0.005 | 0.303 |
| 1-(1-enyl-stearoyl)-2-arachidonoyl-GPE (P-18:0/20:4)* | | Lipid | Plasmalogen | -0.180 | 0.063 | 0.005 | 0.303 |
| proline | | Amino Acid | Urea cycle; Arginine and Proline Metabolism | -0.091 | 0.032 | 0.006 | 0.303 |
| stearoylcarnitine (C18) | | Lipid | Fatty Acid Metabolism (Acyl Carnitine, Long Chain Saturated) | -0.176 | 0.062 | 0.006 | 0.303 |
| glycerol | | Lipid | Glycerolipid Metabolism | 0.196 | 0.070 | 0.006 | 0.303 |
| glyco-beta-muricholate** | | Lipid | Primary Bile Acid Metabolism | -0.408 | 0.145 | 0.007 | 0.303 |
| N-acetylalanine | | Amino Acid | Alanine and Aspartate Metabolism | 0.082 | 0.029 | 0.007 | 0.303 |
| **2,3-dihydroxy-5-methylthio-4-pentenoate (DMTPA)*** | | **Amino Acid** | **Methionine, Cysteine, SAM and Taurine Metabolism** | **0.073** | **0.026** | **0.007** | **0.303** |
| palmitoleate (16:1n7) | | Lipid | Long Chain Monounsaturated Fatty Acid | 0.322 | 0.117 | 0.007 | 0.308 |
| cholic acid glucuronide | | Lipid | Primary Bile Acid Metabolism | 0.464 | 0.169 | 0.009 | 0.332 |
| 1-(1-enyl-stearoyl)-2-oleoyl-GPE (P-18:0/18:1) | | Lipid | Plasmalogen | -0.199 | 0.074 | 0.009 | 0.332 |
| **13-HODE + 9-HODE** | | **Lipid** | **Fatty Acid, Monohydroxy** | **0.201** | **0.075** | **0.009** | **0.339** |
| **linolenate [alpha or gamma; (18:3n3 or 6)]** | | **Lipid** | **Long Chain Polyunsaturated Fatty Acid (n3 and n6)** | **0.255** | **0.097** | **0.010** | **0.345** |
| asparagine | | Amino Acid | Alanine and Aspartate Metabolism | -0.076 | 0.029 | 0.011 | 0.354 |
| myristoleate (14:1n5) | | Lipid | Long Chain Monounsaturated Fatty Acid | 0.311 | 0.121 | 0.012 | 0.393 |
| kynurenine | | Amino Acid | Tryptophan Metabolism | 0.117 | 0.046 | 0.014 | 0.400 |
| phenol sulfate | | Amino Acid | Tyrosine Metabolism | -0.305 | 0.121 | 0.014 | 0.400 |
| quinolinate | | Cofactors and Vitamins | Nicotinate and Nicotinamide Metabolism | 0.190 | 0.076 | 0.014 | 0.400 |
| N-acetyl-3-methylhistidine* | | Amino Acid | Histidine Metabolism | 0.519 | 0.200 | 0.015 | 0.412 |
| 4-acetamidophenol | | Xenobiotics | Drug - Analgesics, Anesthetics | 2.220 | 0.820 | 0.017 | 0.425 |
| methionine sulfoxide | | Amino Acid | Methionine, Cysteine, SAM and Taurine Metabolism | -0.082 | 0.034 | 0.017 | 0.425 |
| N1-Methyl-2-pyridone-5-carboxamide | | Cofactors and Vitamins | Nicotinate and Nicotinamide Metabolism | 0.194 | 0.080 | 0.018 | 0.425 |
| methylsuccinoylcarnitine | | Amino Acid | Leucine, Isoleucine and Valine Metabolism | -0.186 | 0.077 | 0.018 | 0.425 |
| 1-methyl-5-imidazoleacetate | | Amino Acid | Histidine Metabolism | 0.333 | 0.138 | 0.018 | 0.425 |
| androsterone glucuronide | | Lipid | Androgenic Steroids | 0.286 | 0.119 | 0.019 | 0.425 |
| glucuronate | | Carbohydrate | Aminosugar Metabolism | 0.154 | 0.064 | 0.019 | 0.425 |
| sphingomyelin (d18:1/21:0, d17:1/22:0, d16:1/23:0)* | | Lipid | Sphingomyelins | -0.096 | 0.040 | 0.020 | 0.425 |
| sphingomyelin (d18:1/19:0, d19:1/18:0)* | | Lipid | Sphingomyelins | -0.109 | 0.047 | 0.022 | 0.456 |
| pentose acid* | | Partially Characterized Molecules | Partially Characterized Molecules | 0.324 | 0.138 | 0.022 | 0.456 |
| dihomo-linoleate (20:2n6) | | Lipid | Long Chain Polyunsaturated Fatty Acid (n3 and n6) | 0.201 | 0.087 | 0.024 | 0.474 |
| 3-hydroxypyridine glucuronide | | Xenobiotics | Chemical | 1.603 | 0.578 | 0.024 | 0.474 |
| oleate/vaccenate (18:1) | | Lipid | Long Chain Monounsaturated Fatty Acid | 0.210 | 0.092 | 0.025 | 0.475 |
| arabitol/xylitol | | Carbohydrate | Pentose Metabolism | 0.163 | 0.072 | 0.026 | 0.484 |
| N,N,N-trimethyl-5-aminovalerate | | Amino Acid | Lysine Metabolism | -0.129 | 0.057 | 0.027 | 0.484 |
| **N6-carbamoylthreonyladenosine** | | **Nucleotide** | **Purine Metabolism, Adenine containing** | **0.086** | **0.038** | **0.027** | **0.48** |
| 5alpha-pregnan-diol disulfate | | Lipid | Progestin Steroids | 0.391 | 0.175 | 0.029 | 0.497 |
| glycodeoxycholate 3-sulfate | | Lipid | Secondary Bile Acid Metabolism | -0.336 | 0.153 | 0.031 | 0.497 |
| linoleate (18:2n6) | | Lipid | Long Chain Polyunsaturated Fatty Acid (n3 and n6) | 0.159 | 0.072 | 0.031 | 0.497 |
| 1-linoleoyl-2-linolenoyl-GPC (18:2/18:3)* | | Lipid | Phosphatidylcholine (PC) | -0.234 | 0.106 | 0.031 | 0.497 |
| uridine | | Nucleotide | Pyrimidine Metabolism, Uracil containing | 0.124 | 0.056 | 0.032 | 0.497 |
| 3-(3-amino-3-carboxypropyl)uridine* | | Nucleotide | Pyrimidine Metabolism, Uracil containing | 0.094 | 0.043 | 0.032 | 0.497 |
| oleoylcholine | | Lipid | Fatty Acid Metabolism (Acyl Choline) | -0.247 | 0.112 | 0.032 | 0.497 |
| S-1-pyrroline-5-carboxylate | | Amino Acid | Glutamate Metabolism | -0.144 | 0.067 | 0.033 | 0.504 |
| ectoine | | Xenobiotics | Chemical | 0.423 | 0.195 | 0.034 | 0.508 |
| ibuprofen | | Xenobiotics | Drug - Analgesics, Anesthetics | -2.351 | 0.964 | 0.035 | 0.508 |
| methionine | | Amino Acid | Methionine, Cysteine, SAM and Taurine Metabolism | -0.053 | 0.025 | 0.036 | 0.508 |
| arachidonoylcholine | | Lipid | Fatty Acid Metabolism (Acyl Choline) | -0.212 | 0.099 | 0.036 | 0.508 |
| 1-(1-enyl-palmitoyl)-2-linoleoyl-GPC (P-16:0/18:2)* | | Lipid | Plasmalogen | -0.112 | 0.053 | 0.036 | 0.508 |
| myristate (14:0) | | Lipid | Long Chain Saturated Fatty Acid | 0.189 | 0.089 | 0.038 | 0.523 |
| 3-aminoisobutyrate | | Nucleotide | Pyrimidine Metabolism, Thymine containing | 0.211 | 0.103 | 0.043 | 0.579 |
| sphingomyelin (d18:1/20:0, d16:1/22:0)* | | Lipid | Sphingomyelins | -0.066 | 0.032 | 0.047 | 0.628 |

**Supplementary Table S4:** Metabolites linked to higher risk of hypertension in females.

| **Metabolites** | **Super-pathway** | **Sub-pathway** | **Estimate** | **SE** | **p-value** | | **FDR** |
| --- | --- | --- | --- | --- | --- | --- | --- |
| 1-palmitoyl-2-oleoyl-GPE (16:0/18:1) | Lipid | Phosphatidylethanolamine (PE) | 0.296 | 0.079 | | 0.000 | 0.118 |
| 4-acetamidophenol | Xenobiotics | Drug - Analgesics, Anesthetics | 2.229 | 0.568 | | 0.001 | 0.118 |
| 1-linolenoyl-GPC (18:3)* | Lipid | Lysophospholipid | 0.233 | 0.065 | | 0.001 | 0.118 |
| N-palmitoyl-sphingadienine (d18:2/16:0)* | Lipid | Ceramides | 0.192 | 0.054 | | 0.001 | 0.118 |
| gamma-CEHC | Cofactors and Vitamins | Tocopherol Metabolism | 0.291 | 0.083 | | 0.001 | 0.118 |
| 1-arachidonylglycerol (20:4) | Lipid | Monoacylglycerol | 0.283 | 0.085 | | 0.001 | 0.150 |
| oleoyl-linoleoyl-glycerol (18:1/18:2) [1] | Lipid | Diacylglycerol | 0.288 | 0.087 | | 0.001 | 0.150 |
| 1-dihomo-linolenylglycerol (20:3) | Lipid | Monoacylglycerol | 0.238 | 0.072 | | 0.002 | 0.150 |
| 2-hydroxyarachidate* | Lipid | Fatty Acid, Monohydroxy | 0.203 | 0.062 | | 0.002 | 0.150 |
| glycerophosphoethanolamine | Lipid | Phospholipid Metabolism | 0.097 | 0.030 | | 0.002 | 0.150 |
| 1-linoleoylglycerol (18:2) | Lipid | Monoacylglycerol | 0.231 | 0.073 | | 0.002 | 0.156 |
| 1-stearoyl-GPE (18:0) | Lipid | Lysophospholipid | 0.161 | 0.051 | | 0.002 | 0.160 |
| dihomo-linolenate (20:3n3 or n6) | Lipid | Long Chain Polyunsaturated Fatty Acid (n3 and n6) | 0.174 | 0.057 | | 0.003 | 0.187 |
| **N6-carbamoylthreonyladenosine** | **Nucleotide** | **Purine Metabolism, Adenine containing** | **0.094** | **0.031** | | **0.003** | **0.187** |
| cysteinylglycine | Amino Acid | Glutathione Metabolism | 0.190 | 0.063 | | 0.003 | 0.187 |
| adenosine 3',5'-cyclic monophosphate (cAMP) | Nucleotide | Purine Metabolism, Adenine containing | 0.135 | 0.045 | | 0.004 | 0.187 |
| heptenedioate (C7:1-DC)* | Lipid | Fatty Acid, Dicarboxylate | -0.192 | 0.064 | | 0.004 | 0.187 |
| stearate (18:0) | Lipid | Long Chain Saturated Fatty Acid | 0.122 | 0.042 | | 0.004 | 0.187 |
| **stearidonate (18:4n3)** | **Lipid** | **Long Chain Polyunsaturated Fatty Acid (n3 and n6)** | **0.316** | **0.108** | | **0.004** | **0.192** |
| 1-palmitoyl-GPE (16:0) | Lipid | Lysophospholipid | 0.158 | 0.055 | | 0.005 | 0.192 |
| 1-palmitoyl-2-palmitoleoyl-GPC (16:0/16:1)* | Lipid | Phosphatidylcholine (PC) | 0.219 | 0.076 | | 0.005 | 0.192 |
| **2,3-dihydroxy-5-methylthio-4-pentenoate (DMTPA)*** | **Amino Acid** | **Methionine, Cysteine, SAM and Taurine Metabolism** | **0.084** | **0.029** | | **0.005** | **0.193** |
| 2-palmitoyl-GPC (16:0)* | Lipid | Lysophospholipid | 0.178 | 0.063 | | 0.006 | 0.193 |
| 1-oleoyl-GPE (18:1) | Lipid | Lysophospholipid | 0.234 | 0.083 | | 0.006 | 0.193 |
| N-palmitoyl-sphingosine (d18:1/16:0) | Lipid | Ceramides | 0.114 | 0.041 | | 0.006 | 0.194 |
| **hexadecadienoate (16:2n6)** | **Lipid** | **Long Chain Polyunsaturated Fatty Acid (n3 and n6)** | **0.247** | **0.089** | | **0.007** | **0.194** |
| N-acetyl-3-methylhistidine* | Amino Acid | Histidine Metabolism | 0.397 | 0.135 | | 0.007 | 0.194 |
| pseudouridine | Nucleotide | Pyrimidine Metabolism, Uracil containing | 0.076 | 0.028 | | 0.007 | 0.194 |
| behenoyl sphingomyelin (d18:1/22:0)* | Lipid | Sphingomyelins | 0.102 | 0.037 | | 0.007 | 0.194 |
| 12,13-DiHOME | Lipid | Fatty Acid, Dihydroxy | 0.223 | 0.081 | | 0.007 | 0.194 |
| 1-palmitoyl-2-linoleoyl-GPE (16:0/18:2) | Lipid | Phosphatidylethanolamine (PE) | 0.236 | 0.086 | | 0.008 | 0.194 |
| palmitate (16:0) | Lipid | Long Chain Saturated Fatty Acid | 0.140 | 0.051 | | 0.008 | 0.194 |
| EDTA | Xenobiotics | Chemical | 0.068 | 0.025 | | 0.008 | 0.194 |
| oleoyl-linoleoyl-glycerol (18:1/18:2) [2] | Lipid | Diacylglycerol | 0.230 | 0.085 | | 0.008 | 0.194 |
| 1-palmitoyl-2-oleoyl-GPC (16:0/18:1) | Lipid | Phosphatidylcholine (PC) | 0.129 | 0.048 | | 0.008 | 0.194 |
| 3-hydroxybutyroylglycine** | Lipid | Fatty Acid Metabolism (Acyl Glycine) | 0.098 | 0.037 | | 0.009 | 0.211 |
| 1-stearoyl-GPC (18:0) | Lipid | Lysophospholipid | 0.099 | 0.037 | | 0.010 | 0.214 |
| 1-stearoyl-2-oleoyl-GPE (18:0/18:1) | Lipid | Phosphatidylethanolamine (PE) | 0.213 | 0.081 | | 0.010 | 0.214 |
| 1-palmitoyl-GPI (16:0) | Lipid | Lysophospholipid | 0.206 | 0.079 | | 0.010 | 0.214 |
| 1-stearoyl-2-arachidonoyl-GPS (18:0/20:4) | Lipid | Phosphatidylserine (PS) | -0.389 | 0.146 | | 0.010 | 0.214 |
| linoleate (18:2n6) | Lipid | Long Chain Polyunsaturated Fatty Acid (n3 and n6) | 0.169 | 0.065 | | 0.011 | 0.214 |
| 1-palmitoyl-2-arachidonoyl-GPE (16:0/20:4)* | Lipid | Phosphatidylethanolamine (PE) | 0.169 | 0.065 | | 0.011 | 0.214 |
| 1-stearoyl-2-arachidonoyl-GPE (18:0/20:4) | Lipid | Phosphatidylethanolamine (PE) | 0.145 | 0.056 | | 0.011 | 0.217 |
| cholesterol | Lipid | Sterol | 0.081 | 0.032 | | 0.013 | 0.242 |
| oleoyl ethanolamide | Lipid | Endocannabinoid | 0.144 | 0.057 | | 0.013 | 0.242 |
| eugenol sulfate | Xenobiotics | Food Component/Plant | 0.461 | 0.183 | | 0.014 | 0.249 |
| N2,N2-dimethylguanosine | Nucleotide | Purine Metabolism, Guanine containing | 0.095 | 0.038 | | 0.014 | 0.249 |
| cortolone glucuronide (1) | Lipid | Corticosteroids | 0.111 | 0.044 | | 0.014 | 0.249 |
| **13-HODE + 9-HODE** | **Lipid** | **Fatty Acid, Monohydroxy** | **0.178** | **0.071** | | **0.015** | **0.249** |
| 1-linoleoyl-GPE (18:2)* | Lipid | Lysophospholipid | 0.169 | 0.069 | | 0.015 | 0.251 |
| alpha-tocopherol | Cofactors and Vitamins | Tocopherol Metabolism | 0.140 | 0.057 | | 0.016 | 0.251 |
| docosapentaenoate (n3 DPA; 22:5n3) | Lipid | Long Chain Polyunsaturated Fatty Acid (n3 and n6) | 0.211 | 0.086 | | 0.017 | 0.**251** |
| **linolenate [alpha or gamma; (18:3n3 or 6)]** | **Lipid** | **Long Chain Polyunsaturated Fatty Acid (n3 and n6)** | **0.226** | **0.093** | | **0.017** | **0.251** |
| 4-acetamidophenylglucuronide | Xenobiotics | Drug - Analgesics, Anesthetics | 1.387 | 0.557 | | 0.017 | 0.251 |
| 3-(3-amino-3-carboxypropyl)uridine* | Nucleotide | Pyrimidine Metabolism, Uracil containing | 0.103 | 0.043 | | 0.018 | 0.251 |
| N-delta-acetylornithine | Amino Acid | Urea cycle; Arginine and Proline Metabolism | 0.237 | 0.098 | | 0.018 | 0.251 |
| 1-stearoyl-2-oleoyl-GPC (18:0/18:1) | Lipid | Phosphatidylcholine (PC) | 0.145 | 0.060 | | 0.018 | 0.251 |
| oleoylcarnitine (C18:1) | Lipid | Fatty Acid Metabolism (Acyl Carnitine, Monounsaturated) | 0.160 | 0.066 | | 0.018 | 0.251 |
| nisinate (24:6n3) | Lipid | Long Chain Polyunsaturated Fatty Acid (n3 and n6) | 0.236 | 0.097 | | 0.018 | 0.251 |
| N-formylmethionine | Amino Acid | Methionine, Cysteine, SAM and Taurine Metabolism | 0.099 | 0.042 | | 0.020 | 0.268 |
| alpha-CMBHC glucuronide | Cofactors and Vitamins | Tocopherol Metabolism | 0.294 | 0.123 | | 0.020 | 0.268 |
| cys-gly, oxidized | Amino Acid | Glutathione Metabolism | 0.124 | 0.053 | | 0.021 | 0.268 |
| 1-stearoyl-2-linoleoyl-GPE (18:0/18:2)* | Lipid | Phosphatidylethanolamine (PE) | 0.175 | 0.074 | | 0.021 | 0.268 |
| 1-palmitoyl-GPC (16:0) | Lipid | Lysophospholipid | 0.085 | 0.036 | | 0.021 | 0.268 |
| oleate/vaccenate (18:1) | Lipid | Long Chain Monounsaturated Fatty Acid | 0.184 | 0.078 | | 0.021 | 0.268 |
| 1-oleoyl-GPC (18:1) | Lipid | Lysophospholipid | 0.090 | 0.038 | | 0.022 | 0.271 |
| 1-stearoyl-GPI (18:0) | Lipid | Lysophospholipid | 0.148 | 0.063 | | 0.022 | 0.274 |
| 10-heptadecenoate (17:1n7) | Lipid | Long Chain Monounsaturated Fatty Acid | 0.240 | 0.104 | | 0.023 | 0.285 |
| 1-palmitoleoyl-GPC (16:1)* | Lipid | Lysophospholipid | 0.126 | 0.056 | | 0.027 | 0.319 |
| 1-(1-enyl-palmitoyl)-2-linoleoyl-GPC (P-16:0/18:2)* | Lipid | Plasmalogen | 0.109 | 0.049 | | 0.027 | 0.319 |
| 1-myristoyl-2-arachidonoyl-GPC (14:0/20:4)* | Lipid | Phosphatidylcholine (PC) | 0.185 | 0.082 | | 0.027 | 0.319 |
| dihomo-linoleate (20:2n6) | Lipid | Long Chain Polyunsaturated Fatty Acid (n3 and n6) | 0.175 | 0.078 | | 0.028 | 0.319 |
| N-stearoyl-sphingosine (d18:1/18:0)* | Lipid | Ceramides | 0.136 | 0.061 | | 0.028 | 0.319 |
| docosadienoate (22:2n6) | Lipid | Long Chain Polyunsaturated Fatty Acid (n3 and n6) | 0.157 | 0.071 | | 0.029 | 0.325 |
| margarate (17:0) | Lipid | Long Chain Saturated Fatty Acid | 0.140 | 0.063 | | 0.030 | 0.325 |
| 1-myristoyl-2-palmitoyl-GPC (14:0/16:0) | Lipid | Phosphatidylcholine (PC) | 0.204 | 0.092 | | 0.030 | 0.325 |
| suberate (C8-DC) | Lipid | Fatty Acid, Dicarboxylate | 0.153 | 0.069 | | 0.031 | 0.327 |
| 2-linoleoylglycerol (18:2) | Lipid | Monoacylglycerol | 0.202 | 0.091 | | 0.031 | 0.327 |
| palmitoyl sphingomyelin (d18:1/16:0) | Lipid | Sphingomyelins | 0.056 | 0.026 | | 0.032 | 0.334 |
| sarcosine | Amino Acid | Glycine, Serine and Threonine Metabolism | 0.079 | 0.036 | | 0.032 | 0.337 |
| pro-hydroxy-pro | Amino Acid | Urea cycle; Arginine and Proline Metabolism | 0.135 | 0.062 | | 0.033 | 0.342 |
| 1-linolenoylglycerol (18:3) | Lipid | Monoacylglycerol | 0.221 | 0.102 | | 0.034 | 0.345 |
| undecanedioate (C11-DC) | Lipid | Fatty Acid, Dicarboxylate | 0.082 | 0.038 | | 0.035 | 0.352 |
| 1-palmitoyl-2-docosahexaenoyl-GPE (16:0/22:6)* | Lipid | Phosphatidylethanolamine (PE) | 0.164 | 0.077 | | 0.036 | 0.352 |
| sphingomyelin (d18:1/20:0, d16:1/22:0)* | Lipid | Sphingomyelins | 0.072 | 0.034 | | 0.037 | 0.352 |
| alpha-hydroxyisocaproate | Amino Acid | Leucine, Isoleucine and Valine Metabolism | -0.102 | 0.048 | | 0.037 | 0.352 |
| nonadecanoate (19:0) | Lipid | Long Chain Saturated Fatty Acid | 0.086 | 0.041 | | 0.037 | 0.352 |
| pentadecanoate (15:0) | Lipid | Long Chain Saturated Fatty Acid | 0.120 | 0.057 | | 0.037 | 0.352 |
| pregnenolone sulfate | Lipid | Pregnenolone Steroids | -0.213 | 0.101 | | 0.038 | 0.354 |
| 7-alpha-hydroxy-3-oxo-4-cholestenoate (7-Hoca) | Lipid | Sterol | 0.091 | 0.043 | | 0.039 | 0.356 |
| 1-palmitoyl-2-dihomo-linolenoyl-GPC (16:0/20:3n3 or 6)* | Lipid | Phosphatidylcholine (PC) | 0.134 | 0.064 | | 0.039 | 0.356 |
| myristate (14:0) | Lipid | Long Chain Saturated Fatty Acid | 0.169 | 0.081 | | 0.040 | 0.356 |
| (14 or 15)-methylpalmitate (a17:0 or i17:0) | Lipid | Fatty Acid, Branched | 0.142 | 0.068 | | 0.040 | 0.356 |
| 1,2-dipalmitoyl-GPC (16:0/16:0) | Lipid | Phosphatidylcholine (PC) | 0.094 | 0.045 | | 0.041 | 0.362 |
| 3-methylglutaconate | Amino Acid | Leucine, Isoleucine and Valine Metabolism | -0.162 | 0.079 | | 0.042 | 0.362 |
| 1-palmitoyl-2-linoleoyl-GPI (16:0/18:2) | Lipid | Phosphatidylinositol (PI) | 0.132 | 0.064 | | 0.042 | 0.362 |
| 2-stearoyl-GPE (18:0)* | Lipid | Lysophospholipid | 0.148 | 0.072 | | 0.042 | 0.362 |
| 4-cholesten-3-one | Lipid | Sterol | 0.151 | 0.073 | | 0.044 | 0.367 |
| N-acetylalanine | Amino Acid | Alanine and Aspartate Metabolism | 0.067 | 0.033 | | 0.044 | 0.367 |
| N-acetylglucosaminylasparagine | Carbohydrate | Aminosugar Metabolism | 0.081 | 0.040 | | 0.044 | 0.367 |
| gamma-glutamylvaline | Peptide | Gamma-glutamyl Amino Acid | 0.081 | 0.040 | | 0.045 | 0.368 |
| fructose | Carbohydrate | Fructose, Mannose and Galactose Metabolism | -0.072 | 0.036 | | 0.046 | 0.376 |
| N2,N5-diacetylornithine | Amino Acid | Urea cycle; Arginine and Proline Metabolism | 0.172 | 0.085 | | 0.047 | 0.377 |
| 1-linoleoyl-GPC (18:2) | Lipid | Lysophospholipid | 0.086 | 0.043 | | 0.047 | 0.379 |
| 1-arachidonoyl-GPE (20:4n6)* | Lipid | Lysophospholipid | 0.092 | 0.046 | | 0.048 | 0.380 |
| uridine | Nucleotide | Pyrimidine Metabolism, Uracil containing | 0.094 | 0.047 | | 0.048 | 0.380 |

**Supplementary Table S5:** Clinical parameters of the validation cohort categorized by blood pressure. Data are presented as mean (SD), median (IQR) and number (percentage) for parametric, non-parametric and nominal variables respectively. Differences between the groups were tested by ANOVA/Kruskal Wallis for parametric/non-parametric variables and Chi-square test for nominal variables.

|  | **Control** | **Pre-hypertension** | **p-value** |  |
| --- | --- | --- | --- | --- |
| **Number of participants** | 72 | 72 |  |  |
| **Ethnicity** | | Qatari | Qatari |  |
| **Sex (1: Male, 2: Female)** | 1: 61, 2: 11 | 1: 62, 2: 10 | 0.813 |  |
| **Age** | 29 (25-31) | 29 (25-31) | 0.757 |  |
| **BMI** | 25.89 (24.06-27.55) | 26.255 (24.73-27.8) | 0.337 |  |
| **Haemoglobin (g/dl)** | 14.6 (13.7-15.32) | 15 (14-15.45) | 0.230 |  |
| **Haematocrit** | 43.2 (41.35-44.85) | 44.4 (41.9-46.2) | 0.054 |  |
| **Red Blood Cell (x10^6^ ul)** | 5.1 (4.875-5.3) | 5.2 (4.9-5.6) | 0.065 |  |
| **White Blood Cell (x10^3^ ul)** | 5.92 (1.68) | 6.31 (1.53) | 0.146 |  |
| **Platelet (x10^3^ ul)** | 228.78 (51.52) | 231.15 (51.12) | 0.783 |  |
| **Urea (mmol/L)** | 4.9 (3.9-5.7) | 4.75 (3.9-5.52) | 0.818 |  |
| **Glucose (mmol/L)** | 4.93 (0.49) | 4.90 (0.52) | 0.712 |  |
| **Albumin (g/L)** | 46 (45-48) | 47 (45.75-49) | 0.296 |  |
| **ALP** | 68.5 (58.75-79.25) | 69 (56.5-81.5) | 0.900 |  |
| **ALT** | 20 (15-27.25) | 22 (16-33.25) | 0.130 |  |
| **AST** | 18.5 (16-24) | 21 (17.75-24.25) | 0.105 |  |
| **Total Cholesterol (mmol/L)** | 4.5 (4.16-5.00) | 4.58 (4.2-5.3) | 0.490 |  |
| **HDL (mmol/L)** | 1.275 (1.1675-1.55) | 1.28 (1.05-1.43) | 0.200 |  |
| **LDL (mmol/L)** | 2.765 (2.40-3) | 2.96 (2.2975-3.21) | 0.228 |  |
| **Triglyceride (mmol/L)** | 1 (0.64-1.3) | 1 (0.8-1.4) | 0.278 |  |
| **Thyroid Stimulating Hormone** | 1.42 (0.98-2.03) | 1.4 (1.08 -1.98) | 0.786 |  |
| **HBA 1C** | 5.3 (5-5.4) | 5.3 (5.1-5.4) | 0.614 |  |
| **C-Reactive Protein (mg/L)** | 5 (5-5) | 5 (5-5) | 0.674 |  |
| **Systolic Blood Pressure** | 111 (104-115) | 123 (121-127.25) | **3.63E-25** |  |
| **Diastolic Blood Pressure** | 68.71 (6.24) | 77.23 (7.43) | **9.14E-12** |  |
| **Pulse rate Blood Pressure** | 65.11 (8.62) | 66.47 (9.87) | 0.380 |  |
| **History of other comorbidities** |  |  |  |  |
| Diabetes Mellitus | 0 | 0 | 0.999 |  |
| Obesity | 0 | 0 | 0.999 |  |
| Hypercholesterolemia | 8 (11.1%) | 10 (13.8%) | 0.614 |  |
| **Medications** |  |  |  |  |
| Blood pressure medications | 0 | 1 (1.4%) | 0.315 |  |
|  |  |  |  |  |
|  |  |  |  |  |

**Supplementary Table S6:** Metabolites from linear regression model associated with hypertension progression in the randomized discovery and validation cohorts containing diversified age and gender.

| **Metabolites from the discovery cohort including 70% of the participants** | | | | | | |
| --- | --- | --- | --- | --- | --- | --- |
| **Metabolites** | **Sub-pathway** | **Super-pathway** | **Estimate** | **SE** | **p-value** | **FDR** |
| 1-arachidonylglycerol (20:4) | Lipid | Monoacylglycerol | 0.322 | 0.074 | 2.25E-05 | 0.019 |
| linoleate (18:2n6) | Lipid | Long Chain Polyunsaturated Fatty Acid (n3 and n6) | 0.203 | 0.052 | 1.40E-04 | 0.047 |
| palmitate (16:0) | Lipid | Long Chain Saturated Fatty Acid | 0.158 | 0.041 | 1.65E-04 | 0.047 |
| oleoyl-linoleoyl-glycerol (18:1/18:2) [1] | Lipid | Diacylglycerol | 0.299 | 0.079 | 2.20E-04 | 0.047 |
| **linolenate [alpha or gamma; (18:3n3 or 6)]** | **Lipid** | **Long Chain Polyunsaturated Fatty Acid (n3 and n6)** | **0.269** | **0.073** | **2.84E-04** | **0.048** |
| **stearidonate (18:4n3)** | **Lipid** | **Long Chain Polyunsaturated Fatty Acid (n3 and n6)** | **0.303** | **0.084** | **3.65E-04** | **0.052** |
| docosadienoate (22:2n6) | Lipid | Long Chain Polyunsaturated Fatty Acid (n3 and n6) | 0.206 | 0.058 | 4.52E-04 | 0.052 |
| N-acetylcarnosine | Amino Acid | Histidine Metabolism | 0.164 | 0.046 | 4.86E-04 | 0.052 |
| dihomo-linoleate (20:2n6) | Lipid | Long Chain Polyunsaturated Fatty Acid (n3 and n6) | 0.219 | 0.063 | 6.20E-04 | 0.055 |
| 1-linoleoylglycerol (18:2) | Lipid | Monoacylglycerol | 0.214 | 0.062 | 7.07E-04 | 0.055 |
| oleate/vaccenate (18:1) | Lipid | Long Chain Monounsaturated Fatty Acid | 0.223 | 0.065 | 7.14E-04 | 0.055 |
| **hexadecadienoate (16:2n6)** | **Lipid** | **Long Chain Polyunsaturated Fatty Acid (n3 and n6)** | **0.246** | **0.075** | **1.28E-03** | **0.081** |
| 1-palmitoyl-GPI (16:0) | Lipid | Lysophospholipid | 0.198 | 0.061 | 1.38E-03 | 0.081 |
| pentadecanoate (15:0) | Lipid | Long Chain Saturated Fatty Acid | 0.150 | 0.046 | 1.40E-03 | 0.081 |
| eicosenoate (20:1) | Lipid | Long Chain Monounsaturated Fatty Acid | 0.207 | 0.064 | 1.48E-03 | 0.081 |
| 1-palmitoyl-2-oleoyl-GPC (16:0/18:1) | Lipid | Phosphatidylcholine (PC) | 0.112 | 0.035 | 1.58E-03 | 0.081 |
| stearate (18:0) | Lipid | Long Chain Saturated Fatty Acid | 0.110 | 0.034 | 1.67E-03 | 0.081 |
| 1-palmitoyl-2-palmitoleoyl-GPC (16:0/16:1)* | Lipid | Phosphatidylcholine (PC) | 0.180 | 0.057 | 1.76E-03 | 0.081 |
| myristate (14:0) | Lipid | Long Chain Saturated Fatty Acid | 0.202 | 0.064 | 1.81E-03 | 0.081 |
| 1-stearoyl-GPI (18:0) | Lipid | Lysophospholipid | 0.155 | 0.049 | 1.91E-03 | 0.081 |
| 1-dihomo-linolenylglycerol (20:3) | Lipid | Monoacylglycerol | 0.212 | 0.067 | 2.00E-03 | 0.081 |
| 1,2-dipalmitoyl-GPC (16:0/16:0) | Lipid | Phosphatidylcholine (PC) | 0.105 | 0.034 | 2.08E-03 | 0.081 |
| docosapentaenoate (n3 DPA; 22:5n3) | Lipid | Long Chain Polyunsaturated Fatty Acid (n3 and n6) | 0.206 | 0.067 | 2.28E-03 | 0.084 |
| 4-acetamidophenol | Xenobiotics | Drug - Analgesics, Anesthetics | 1.493 | 0.471 | 2.51E-03 | 0.086 |
| margarate (17:0) | Lipid | Long Chain Saturated Fatty Acid | 0.160 | 0.052 | 2.52E-03 | 0.086 |
| oleoyl-linoleoyl-glycerol (18:1/18:2) [2] | Lipid | Diacylglycerol | 0.224 | 0.074 | 2.91E-03 | 0.094 |
| docosahexaenoate (DHA; 22:6n3) | Lipid | Long Chain Polyunsaturated Fatty Acid (n3 and n6) | 0.167 | 0.056 | 2.99E-03 | 0.094 |
| palmitoleate (16:1n7) | Lipid | Long Chain Monounsaturated Fatty Acid | 0.262 | 0.089 | 3.42E-03 | 0.099 |
| 1-palmitoyl-2-oleoyl-GPE (16:0/18:1) | Lipid | Phosphatidylethanolamine (PE) | 0.195 | 0.066 | 3.58E-03 | 0.099 |
| arachidonate (20:4n6) | Lipid | Long Chain Polyunsaturated Fatty Acid (n3 and n6) | 0.123 | 0.042 | 3.77E-03 | 0.099 |
| 10-heptadecenoate (17:1n7) | Lipid | Long Chain Monounsaturated Fatty Acid | 0.230 | 0.079 | 3.79E-03 | 0.099 |
| **13-HODE + 9-HODE** | **Lipid** | **Fatty Acid, Monohydroxy** | **0.156** | **0.054** | **3.94E-03** | **0.099** |
| 1-linoleoyl-GPI (18:2)* | Lipid | Lysophospholipid | 0.149 | 0.051 | 3.99E-03 | 0.099 |
| 1-palmitoyl-2-linoleoyl-GPI (16:0/18:2) | Lipid | Phosphatidylinositol (PI) | 0.132 | 0.046 | 4.14E-03 | 0.099 |
| 3beta-hydroxy-5-cholestenoate | Lipid | Sterol | 0.156 | 0.054 | 4.14E-03 | 0.099 |
| 1-linolenoyl-GPC (18:3)* | Lipid | Lysophospholipid | 0.151 | 0.052 | 4.17E-03 | 0.099 |

| **Metabolites from the validation cohort including 30% of the participants** | | | | | | |
| --- | --- | --- | --- | --- | --- | --- |
| **Metabolites** | **Sub-pathway** | **Super-pathway** | **Estimate** | **SE** | **p-value** | **FDR** |
| N2,N5-diacetylornithine | Amino Acid | Urea cycle; Arginine and Proline Metabolism | 0.329 | 0.106 | 0.003 | 0.995 |
| adenosine 3',5'-cyclic monophosphate (cAMP) | Nucleotide | Purine Metabolism, Adenine containing | 0.136 | 0.051 | 0.010 | 0.995 |
| **stearidonate (18:4n3)** | **Lipid** | **Long Chain Polyunsaturated Fatty Acid (n3 and n6)** | **0.337** | **0.131** | **0.012** | **0.995** |
| 3-aminoisobutyrate | Nucleotide | Pyrimidine Metabolism, Thymine containing | 0.236 | 0.094 | 0.014 | 0.995 |
| 4-imidazoleacetate | Amino Acid | Histidine Metabolism | 0.234 | 0.093 | 0.015 | 0.995 |
| pantothenate | Cofactors and Vitamins | Pantothenate and CoA Metabolism | 0.193 | 0.079 | 0.017 | 0.995 |
| hydroxy-N6,N6,N6-trimethyllysine* | Amino Acid | Lysine Metabolism | 0.144 | 0.060 | 0.018 | 0.995 |
| methylsuccinate | Amino Acid | Leucine, Isoleucine and Valine Metabolism | 0.159 | 0.066 | 0.018 | 0.995 |
| isovalerate (i5:0) | Amino Acid | Leucine, Isoleucine and Valine Metabolism | -0.134 | 0.056 | 0.019 | 0.995 |
| gamma-glutamylglycine | Peptide | Gamma-glutamyl Amino Acid | 0.124 | 0.052 | 0.019 | 0.995 |
| 4-methyl-2-oxopentanoate | Amino Acid | Leucine, Isoleucine and Valine Metabolism | -0.123 | 0.052 | 0.019 | 0.995 |
| ascorbic acid 2-sulfate | Cofactors and Vitamins | Ascorbate and Aldarate Metabolism | 0.143 | 0.060 | 0.019 | 0.995 |
| **hexadecadienoate (16:2n6)** | **Lipid** | **Long Chain Polyunsaturated Fatty Acid (n3 and n6)** | **0.274** | **0.119** | **0.023** | **0.995** |
| 2-hydroxyhippurate (salicylurate) | Xenobiotics | Benzoate Metabolism | 0.381 | 0.166 | 0.024 | 0.995 |
| 2-hydroxy-3-methylvalerate | Amino Acid | Leucine, Isoleucine and Valine Metabolism | -0.177 | 0.078 | 0.025 | 0.995 |
| glucuronate | Carbohydrate | Aminosugar Metabolism | 0.141 | 0.064 | 0.030 | 0.995 |
| N-delta-acetylornithine | Amino Acid | Urea cycle; Arginine and Proline Metabolism | 0.235 | 0.107 | 0.031 | 0.995 |
| 1-methyl-4-imidazoleacetate | Amino Acid | Histidine Metabolism | 0.117 | 0.054 | 0.032 | 0.995 |
| tyrosine | Amino Acid | Tyrosine Metabolism | -0.096 | 0.044 | 0.033 | 0.995 |
| acetylcarnitine (C2) | Lipid | Fatty Acid Metabolism (Acyl Carnitine, Short Chain) | -0.389 | 0.181 | 0.035 | 0.995 |
| retinol (Vitamin A) | Cofactors and Vitamins | Vitamin A Metabolism | 0.102 | 0.048 | 0.037 | 0.995 |
| alpha-hydroxyisovalerate | Amino Acid | Leucine, Isoleucine and Valine Metabolism | -0.156 | 0.075 | 0.039 | 0.995 |
| phenol sulfate | Amino Acid | Tyrosine Metabolism | -0.214 | 0.106 | 0.046 | 0.995 |
| 5-HETE | Lipid | Eicosanoid | -0.155 | 0.077 | 0.047 | 0.995 |
| glycerol | Lipid | Glycerolipid Metabolism | 0.139 | 0.070 | 0.048 | 0.995 |
| 1-ribosyl-imidazoleacetate* | Amino Acid | Histidine Metabolism | 0.126 | 0.063 | 0.049 | 0.995 |
